# Proteomic Profiling of the TRAF3 Interactome Network Reveals a New Role for the ER-to-Golgi Transport Compartments in Innate Immunity

**DOI:** 10.1371/journal.ppat.1002747

**Published:** 2012-07-05

**Authors:** Wendy J. van Zuylen, Priscilla Doyon, Jean-François Clément, Kashif Aziz Khan, Lisa M. D'Ambrosio, Florence Dô, Myriam St-Amant-Verret, Tasheen Wissanji, Gregory Emery, Anne-Claude Gingras, Sylvain Meloche, Marc J. Servant

**Affiliations:** 1 Faculty of Pharmacy, Université de Montréal, Montréal, Québec Canada; 2 Samuel Lunenfeld Research Institute at Mount Sinai Hospital, Toronto, Ontario, Canada; Department of Molecular Genetics, University of Toronto, Toronto, Ontario, Canada; 3 Institut de Recherche en Immunologie et Cancérologie, Université de Montréal, Montréal, Québec, Canada; 4 Department of Pathology and Cell Biology, Université de Montréal, Montréal, Québec, Canada; 5 Departments of Pharmacology and Molecular Biology, Université de Montréal, Montréal, Québec Canada; Kantonal Hospital St. Gallen, Switzerland

## Abstract

Tumor Necrosis Factor receptor-associated factor-3 (TRAF3) is a central mediator important for inducing type I interferon (IFN) production in response to intracellular double-stranded RNA (dsRNA). Here, we report the identification of Sec16A and p115, two proteins of the ER-to-Golgi vesicular transport system, as novel components of the TRAF3 interactome network. Notably, in non-infected cells, TRAF3 was found associated with markers of the ER-Exit-Sites (ERES), ER-to-Golgi intermediate compartment (ERGIC) and the cis-Golgi apparatus. Upon dsRNA and dsDNA sensing however, the Golgi apparatus fragmented into cytoplasmic punctated structures containing TRAF3 allowing its colocalization and interaction with Mitochondrial AntiViral Signaling (MAVS), the essential mitochondria-bound RIG-I-like Helicase (RLH) adaptor. In contrast, retention of TRAF3 at the ER-to-Golgi vesicular transport system blunted the ability of TRAF3 to interact with MAVS upon viral infection and consequently decreased type I IFN response. Moreover, depletion of Sec16A and p115 led to a drastic disorganization of the Golgi paralleled by the relocalization of TRAF3, which under these conditions was unable to associate with MAVS. Consequently, upon dsRNA and dsDNA sensing, ablation of Sec16A and p115 was found to inhibit IRF3 activation and anti-viral gene expression. Reciprocally, mild overexpression of Sec16A or p115 in Hec1B cells increased the activation of IFNβ, ISG56 and NF-κB -dependent promoters following viral infection and ectopic expression of MAVS and Tank-binding kinase-1 (TBK1). In line with these results, TRAF3 was found enriched in immunocomplexes composed of p115, Sec16A and TBK1 upon infection. Hence, we propose a model where dsDNA and dsRNA sensing induces the formation of membrane-bound compartments originating from the Golgi, which mediate the dynamic association of TRAF3 with MAVS leading to an optimal induction of innate immune responses.

## Author Summary

In response to pathogens, such as viruses and bacteria, infected cells defend themselves by generating a set of cytokines called type I interferon (IFN). Since Type I IFN (namely IFN alpha and beta) are potent antiviral agents, understanding the cellular mechanisms by which infected cells produce type I IFN is required to identify novel cellular targets for future antiviral therapies. Notably, a protein called Tumor Necrosis Factor receptor-associated factor-3 (TRAF3) was demonstrated to be an essential mediator of this antiviral response. However, how TRAF3 reacts in response to a viral infection is still not totally understood. We now demonstrate that, through its capacity to interact with other proteins (namely Sec16A and p115) that normally control protein secretion, TRAF3 resides close to the nucleus in uninfected cells, in a region called the ER-to-Golgi Intermediate Compartment (ERGIC). Following viral infection, the ERGIC reorganizes into small punctate structures allowing TRAF3 to associate with Mitochondrial AntiViral Signaling (MAVS), an essential adaptor of the anti-viral type I IFN response. Thus, our study reveals an unpredicted role of the protein secretion system for the proper localization of TRAF3 and the antiviral response.

## Introduction

Following exposure to pathogen-associated molecular patterns (PAMPs), the innate immune response and the subsequent inflammatory reaction rely on evolutionarily conserved receptors termed pattern-recognition receptors (PRRs) [1]. These signalling receptors can be expressed at the cellular membrane (Toll-like receptors (TLRs) 1, 2, 4, 5, and 6), in acidic endosomes (TLRs 3, 7, 8, and 9), or in the cytoplasmic compartment (the double-stranded RNA (dsRNA)-activated kinase (PKR); the RIG-I-like helicases (RLH): retinoic-acid-inducible gene I (RIG-I), melanoma differentiation antigen 5 (MDA5), and LGP2; the HIN-200 family members: Absent In Melanoma 2 (AIM2) and interferon (IFN)-inducible IFI16 protein [2]; the DNA-dependent activator of interferon regulatory factors (IRFs) (DAI) and the nucleotide-binding oligomerization domain (NOD) receptors). RIG-I and MDA5 have been characterized as important cytoplasmic sensors for viral RNA [3]–[6]. Once activated by dsRNA molecules, RIG-I and MDA5 are recruited to the mitochondrial adaptor protein know as Mitochondrial AntiViral Signaling (MAVS) (also called IPS-1, Cardif and VISA) in order to trigger signalling cascades leading to IRF-3 and NF-κB activation, two essential players involved in the establishment of a cellular antiviral state [7]–[10].

Tumor Necrosis Factor (TNF) receptor-associated factors (TRAFs) are part of a family of adaptor proteins that bridge the intracellular domains of multiple receptors, such as TNFR, IL1R, and TLRs, to downstream effectors involved in the inflammatory and innate immune signalling pathways. The TRAF family is composed of seven members, TRAF1 through TRAF7. They all share a C-terminal TRAF domain, which is composed of a coiled-coil domain followed by a conserved receptor-interacting domain. This domain mediates self-association and interaction with receptors or signalling proteins. Their N-terminal regions are composed of one or more zinc-finger motifs and, with the exception of TRAF1, a RING-finger domain that mediates E3 ubiquitin ligase activity and signalling [11]. All mammalian TRAFs localize to the cytoplasm except TRAF4, which is found in the nucleus. Importantly, gene deletion studies have identified TRAF3 as a critical mediator involved in the induction of the type I interferons (IFNs) by the RLH pathway [12], [13].

TRAF3 has originally been shown to associate with TNF receptors (e.g. BAFFR, CD40, LTβR, RANK, CD30, and Fn14), which are activators of the non-canonical NF-κB pathway [14]–[17]. TRAF3 acts as a negative regulator in this pathway by promoting the recruitment of the TRAF2-cIAP1-cIAP2 E3 ligase complex to NF-κB-inducing kinase (NIK) in order to control its rapid turnover in resting cells [18], [19]. However, in the RLH pathway, the adaptor protein TRAF3 acts as a positive regulator. Its interaction with MAVS and TRADD is important to trigger IRF-3 phosphorylation through the adaptor molecule TANK and the IKK-related kinases TBK1 and IKKi [20], [21]. The TRADD-mediated recruitment of FADD and RIP1 to MAVS also enhances the interaction between TANK and TRAF3. A model was then proposed in which TRADD simultaneously organizes FADD- and RIP1-mediated NF-κB signalling on one hand and TRAF3- and TANK-mediated IRF-3 signalling on the other [21], [22]. However, this possible mechanism of action requires further investigation to determine how TRAF3 is recruited to the mitochondrial adaptor protein MAVS upon viral infection.

Here, we have used a proteomics-based strategy to identify novel TRAF3 interacting proteins that are implicated in the induction of type I IFN. Using this approach, we have identified two novel TRAF3 interactors, Sec16A (also known as KIAA0310) and p115 (also known as USO1), which have characterized roles in the Endoplasmic Reticulum (ER)-to-Golgi vesicular transport system. Both proteins were shown to play a primary role in the anterograde trafficking at the ER-Golgi interface by influencing the assembly and transport of coat protein complex II (COPII) vesicles. Sec16A assembles on the ER membrane and forms organized scaffold defining ER exit sites (ERES) where COPII assembly occurs [23]–[25]. The coiled-coil myosin-shaped molecule p115 was demonstrated to be an important tethering adaptor, which mediates vesicle tethering at the ER [26], Endoplasmatic Reticulum-Golgi Intermediate Compartment (ERGIC) [27], and in conjunction with tether proteins giantin and GM130 at the cis-Golgi [28], [29].

Since novel essential mediators of the type I IFN response were recently found to be associated with the ER or the exocyst pathway, such as STING (also called MITA, ERIS, and MPYS), Sec61β and Sec5 [30]–[33], we postulated that Sec16A and p115 may exert a similar function through the ER-to-Golgi transport compartments. Co-immunoprecipitation experiments and confocal microscopy confirmed the association and co-localization of TRAF3 with p115 and Sec16A. Importantly, overexpression of p115 or Sec16A increased the type I IFN response, whereas their knockdown impaired the induction of antiviral genes. Interestingly, the Golgi apparatus fragmented into cytoplasmic punctate structures following both RLH and cytoplasmic DNA sensor pathway activation, allowing TRAF3 to colocalize and associate with MAVS. Our study identifies p115 and Sec16A as new scaffold proteins involved in the establishment of the antiviral state.

## Results

### Identification of Sec16A and p115 as new TRAF3 interactors

**Figure 1:**
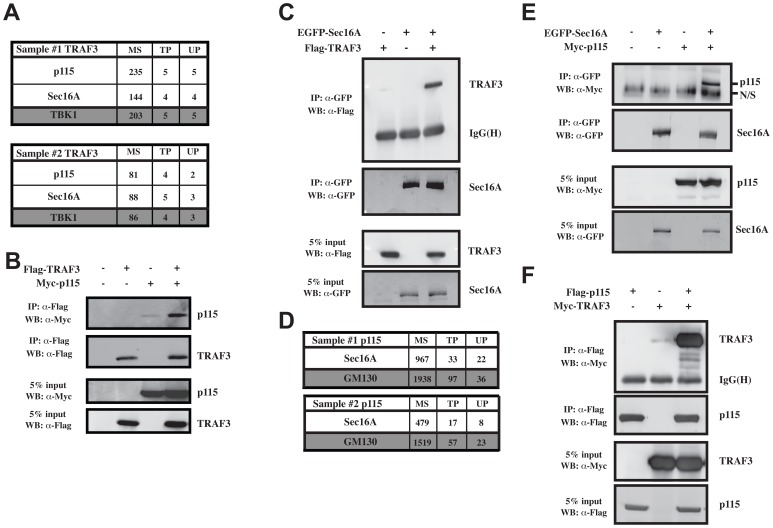
Identification of p115 and Sec16A as new TRAF3 interacting proteins. (**A**) HEK293 cells were stably transfected with pcDNA3-FLAG-TRAF3 or pcDNA3-FLAG alone. After G418 selection, the cells were lysed and subjected to AP/MS as described in Methods. The complete list of interactors is shown in Figure S1A. Data for p115 and Sec16A, which were not detected in immunoprecipitates of control cells, are shown here. MS; mascot score, TP; average total number of peptides (spectral counts) identified, UP; number of unique peptides observed; data from two biological replicates are shown. (**B**) Co-immunoprecipitation experiments showing the association of Myc-p115 to FLAG-TRAF3 when coexpressed in 293T cells. One of three independent experiments with similar results is shown. (**C**) Co-immunoprecipitation experiments showing the association of FLAG-TRAF3 to EGFP-Sec16A when coexpressed in 293T cells. One of three independent experiments with similar results is shown. (**D**) AP/MS analysis from cells stably transfected with pcDNA3-FLAG-p115 or pcDNA3-FLAG vector and analyzed as described in A). (**E**) Co-immunoprecipitation experiments showing the association of Myc-p115 to EGFP-Sec16A and (**F**) Myc-TRAF3 to FLAG-p115 when coexpressed in 293T cells. One of three independent experiments with similar results is shown.

In order to find novel players involved in the type I IFN pathway, we have used a functional proteomics approach based on FLAG affinity purification and mass spectrometry analysis (AP/MS). HEK293 cells stably expressing FLAG-TRAF3 were harvested, subjected to IP with an anti-FLAG antibody under native conditions and FLAG-TRAF3 complexes were analyzed by liquid chromatography coupled to tandem mass spectrometry (LC-MS/MS). In parallel, multiple AP/MS analyses were performed from cells expressing the FLAG alone. Following standard database searches, stringent statistical filtering was performed using SAINT (see Methods). Proteins detected with AvgP≥0.7 were manually inspected for frequency of detection across a database of ∼1000 AP/MS analyses, and proteins frequently detected in AP-MS experiments were removed. This resulted in the identification of 12 interaction partners for TRAF3, including TBK1, a well-known TRAF3 interactor [12], [13]. Surprisingly, Sec16A and p115, two proteins involved in ER-to-Golgi vesicular trafficking, were found to associate with TRAF3 immunocomplexes with a high confidence (Figure 1A and Figure S1).

To confirm these interactions, we performed conventional co-immunoprecipitation experiments by overexpressing the candidate tagged-proteins with FLAG-TRAF3 in 293T cells. The interactions between FLAG-TRAF3 and Myc-p115 or EGFP-Sec16A were clearly detected (Figure 1B–C). To further substantiate the interaction network between TRAF3, Sec16A and p115, we additionally established a pool of HEK293 cells stably expressing FLAG-p115 and analyzed its physiological interactors by FLAG affinity purification and LC-MS/MS, followed by analysis with SAINT. FLAG-p115 was found to be associated with nine proteins (after filtering), including Sec16A and GM130 (also known as GOLGA2), an established physical partner of p115 [34] (Figure 1D and Figure S1). The interaction of EGFP-Sec16A with Myc-p115 was further confirmed by co-immunoprecipitation experiments (Figure 1E). However, endogenous TRAF3 was not recovered in our FLAG-p115 analysis. This result may be explained by the fact that p115 has many higher-abundance interactors and/or is part of alternative complexes independent from TRAF3. However, overexpressed Myc-TRAF3 was recovered from FLAG-p115 complexes when the latter were immunoprecipitated from 293T cells co-expressing both constructs (Figure 1F). The TRAF3 interactome network identified by functional proteomics (Figure S1B) suggests the presence of at least a fraction of TRAF3 in close proximity to the Golgi network.

Members of the TRAF family often share common interacting partners. For example, TRADD and RIP1 strongly bind to TRAF1, TRAF2 and TRAF3 [21], [35], whereas the mitochondrial anti-viral signaling protein MAVS interacts with TRAF2, TRAF3 and TRAF6 [10], [20]. To verify the binding selectivity of the newly identified TRAF3 interactors, we next performed co-immunoprecipitation experiments in 293T cells overexpressing FLAG-tagged TRAF2, TRAF3 or TRAF6, TRAF molecules involved in type I IFN and inflammatory responses, along with Myc-p115 or EGFP-Sec16A. Only Myc-p115 was found to be enriched in FLAG-TRAF3 immunocomplexes (Figure S1C). A similar result was obtained with EGFP-Sec16A, except that a weak enrichment was observed with TRAF2 when compared to TRAF3 (Figure S1D).

### TRAF3 localizes to the ER-to-Golgi transport compartments and behaves like a cis-Golgi matrix protein

**Figure 2:**
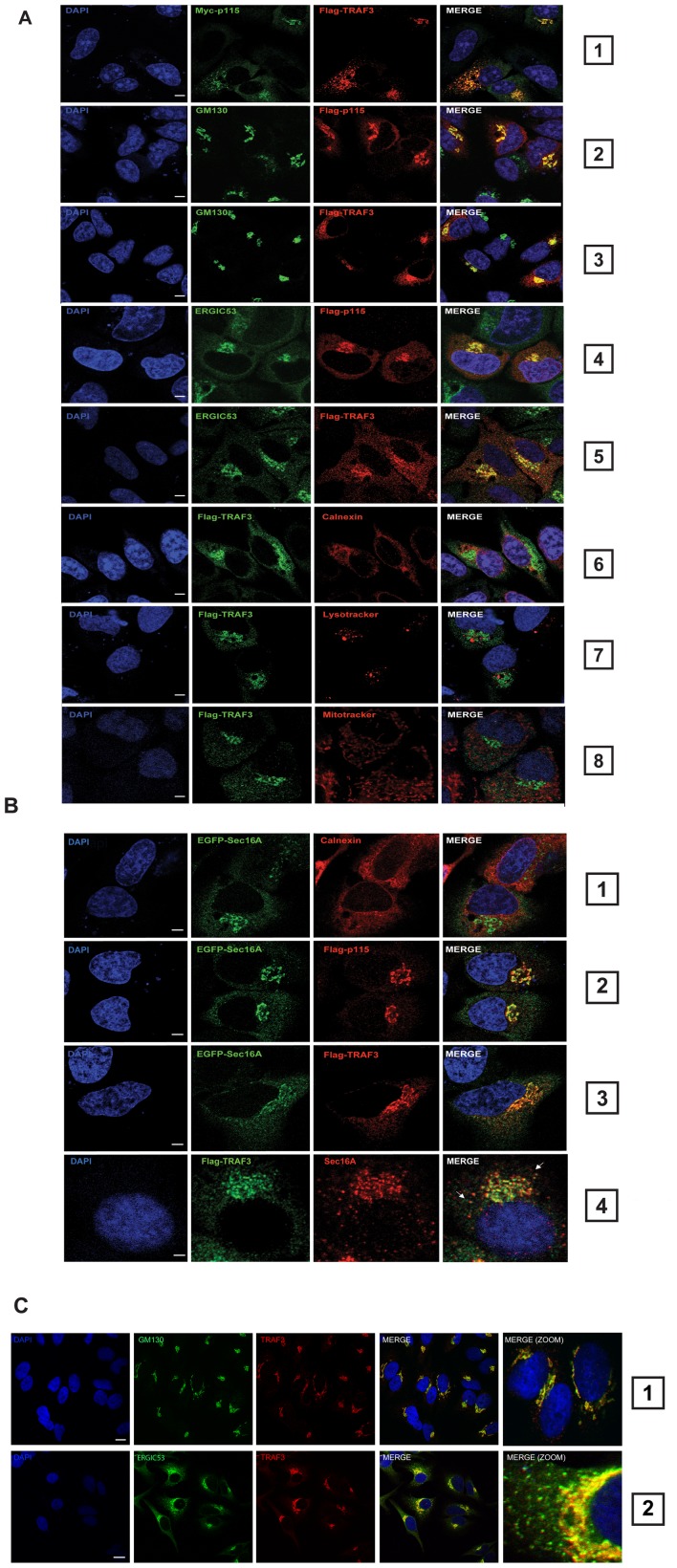
TRAF3 localizes to the ER-to-Golgi transport compartments and behaves like a cis-Golgi protein. (**A**) Confocal microscopy performed in HeLa cells on FLAG-TRAF3 and Myc-p115 (panel 1), FLAG-p115 and GM130 (panel 2), FLAG-TRAF3 and GM130 (panel 3), FLAG-p115 and ERGIC53 (panel 4), FLAG-TRAF3 and ERGIC53 (panel 5), FLAG-TRAF3 and Calnexin (panel 6), FLAG-TRAF3 and lysotracker (panel 7) or mitotracker (panel 8). The nuclei were stained utilizing DAPI. One of three independent experiments with similar results is shown. Bars represent 10 µm. (**B**) Confocal microscopy performed in HeLa cells on EGFP-Sec16A and calnexin (panel 1), EGFP-Sec16A and FLAG-p115 (panel 2), EGFP-Sec16A and FLAG-TRAF3 (panel 3) and FLAG-TRAF3 and endogenous Sec16A (panel 4). (**C**) HeLa cells were stained for endogenous TRAF3 and GM130 (panel 1) or endogenous TRAF3 and ERGIC53 (panel 2) before analysis via confocal microscopy. The nuclei were stained utilizing DAPI. Bars represent 5 µm. One of three independent experiments with similar results is shown.

To further validate the interaction between TRAF3 and these new interactors, we next analyzed their subcellular localization by confocal microscopy. The vesicle-tethering protein p115 is known to colocalize and interact specifically with the NH2 terminus of the cis-Golgi protein GM130 ([36] and see Figure 2A, panel 2). Upon ectopic expression of Myc-p115 and FLAG-TRAF3, we observed a co-localization of these two proteins (Figure 2A, panel 1). FLAG-TRAF3 was also observed to localize to the Golgi apparatus where it exhibits a high degree of overlap with the cis-Golgi marker GM130 (Figure 2A, panel 3). p115 was previously reported to be present in the ERGIC, through an interaction involving activated Rab1 [37], [38]. This cellular localization of FLAG-p115 can be visualized with the conventional ERGIC marker, ERGIC53 (Figure 2A panel 4). Notably, FLAG-TRAF3 (or Myc-TRAF3 (unpublished data)) was also present in the ERGIC (Figure 2A, panel 5). No significant colocalization was detected between TRAF3 and the ER marker calnexin (Figure 2A, panel 6), the lysosomal compartments (Figure 2A, panel 7) and the mitochondrial network (Figure 2A, panel 8).

In HeLa cells, Sec16A was demonstrated to define ERES [24], [25], localizing to punctate structures on the ER membrane. This pattern was reproduced in this study (Figure 2B, panel 1). Since Rab1 recruitment of p115 to ERES [26] represents an essential step for the subsequent docking of ER-derived vesicles to the ERGIC [39], we next examined the colocalization of EGFP-Sec16A and FLAG-p115. The two proteins clearly colocalized at the perinuclear region (Figure 2B, panel 2). FLAG-TRAF3 and EGFP-Sec16A also mainly colocalized at the perinuclear region in HeLa cells (Figure 2B, panel 3). Moreover, a colocalization of FLAG-TRAF3 with endogenous Sec16A at ERES distributed in the cytoplasm could be observed. However, some FLAG-TRAF3 punctae also appeared in close proximity to those containing Sec16A (Figure 2B, panel 4, compare arrows). Ectopic expression of FLAG-TRAF2 and FLAG-TRAF6 revealed that only TRAF3 exhibits a cellular Golgi-like distribution (Figure S2A) and colocalizes with endogenous Sec16A (Figure S2B) or Myc-p115 (Figure S2C). Importantly, endogenous staining of TRAF3 revealed that the majority of TRAF3 proteins localized to the juxtanuclear region containing both the cis-Golgi marker GM130 and the ERGIC marker ERGIC53 (Figure 2C).

To further confirm the localization of TRAF3 to the Golgi apparatus, we next treated the cells with nocodazole. Microtubule depolymerization is known to result in the reorganization of the Golgi complex into characteristic mini-stacks, which appear as punctate structures throughout the cell [40]. Nevertheless, FLAG-TRAF3 was detected to colocalize with GM130 and Myc-p115 in cells treated with nocodazole (Figure S3, panels 1, 2 and 3). Treatment with brefeldin A (BFA), leads to relocalization of the components of the cis-Golgi matrix to cytoplasmic punctate structures (also called remnants) that appear close to ERES [41], [42]. GM130 and p115 are cis-Golgi proteins, which are known to be relocalized to these remnants [41]. FLAG-TRAF3 was also relocated to cytoplasmic remnants upon BFA treatment, where it co-localized with GM130 and Myc-p115 (Figure S3, panels 4 and 5). Altogether, results from our pharmacological experiments and confocal microscopy strongly suggest that TRAF3 localized to ER-to-Golgi transport compartments, where it tightly associates [43], [44].

### TRAF3 localization and interaction with components of the ER-to-Golgi vesicular pathway requires its protein integrity

**Figure 3:**
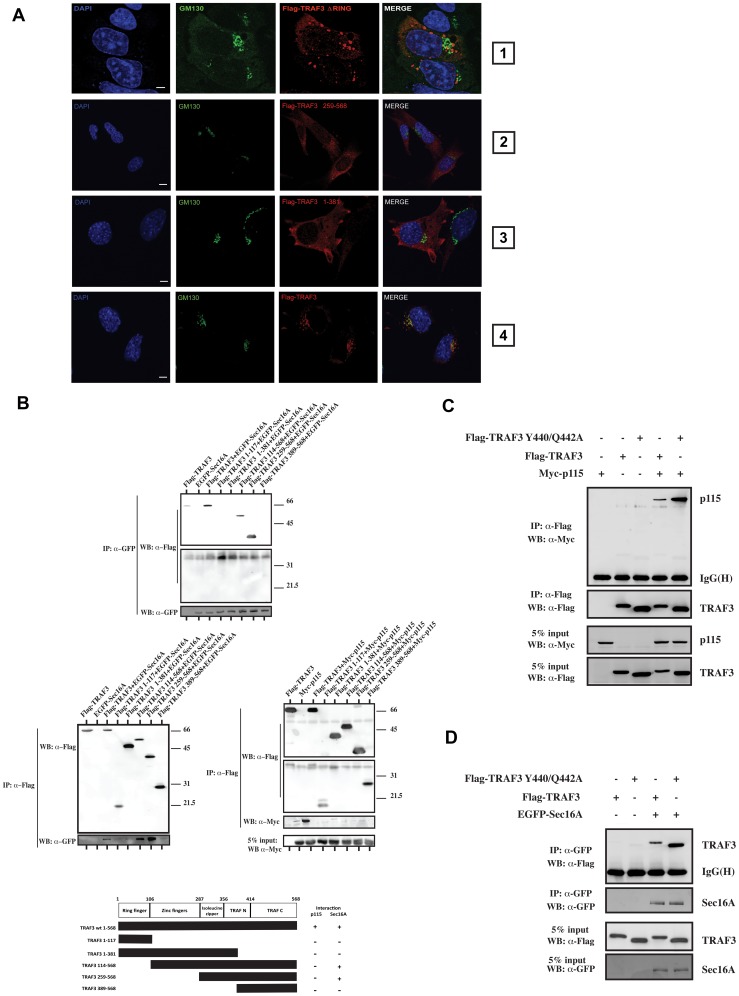
TRAF3 interaction with sec16A or p115 requires its protein integrity. (**A**) Confocal microscopy analysis of HeLa cells expressing FLAG-TRAF3 deltaRING (panel 1). Additionally, TRAF3 knockout MEF cells were transfected with FLAG-TRAF3 259–568 (panel 2), FLAG-TRAF3 1–381 (panel 3), or FLAG-TRAF3 WT (panel 4) and stained for GM130, FLAG-tag, and DAPI (nucleus) before analysis via confocal microscopy. Bars represent 5 µm. One of three independent experiments with similar results is shown. (**B**) Co-immunoprecipitation experiments showing the association of EGFP-Sec16A and Myc-p115 to FLAG-TRAF3 deletion mutants when coexpressed in 293T cells (upper and lower parts from the Western blot are from the same gel). One of three independent experiments with similar results is shown. The bottom panel is a linear representation of TRAF3 deletion mutants and their capacity to interact with p115 or Sec16A. Numbers indicate the position of amino acids in TRAF3. (**C–D**) Co-immunoprecipitation experiments showing the association of FLAG-TRAF3 WT and Y440/Q442A with Myc-p115 or EGFP-Sec16A immunocomplexes when coexpressed in 293T cells.

It has been proposed that a structurally intact TRAF3 molecule is required for its biological function. Indeed, TRAF3 lacking its N-terminal RING or the C-terminal TRAF domain lacks antiviral activity [20]. We therefore examined the subcellular localization of TRAF3 deletion mutants in reconstituted TRAF3 knockout MEF cells. Removal of the N-terminal Ring Finger domain (Figure 3A, panel 1), the N-terminal Ring and Zinc finger domains (Figure 3A, panel 2) or the C-terminal TRAF domain (Figure 3A, panel 3) resulted in TRAF3 molecules that no longer colocalize with the Golgi marker GM130. Furthermore, coimmunoprecipitation experiments in 293T cells revealed that immunocomplexes containing p115 are detected only with full length TRAF3 and that Sec16A-containing immunocomplexes required at least the isoleucine zipper and the TRAF domain (Figure 3B). Moreover, TRAF3 is known to interact with several substrates containing a particular motif (PxQxS/T) called the TRAF interaction motif (TIM) [45]. The mutation of two amino acids located in the TIM-binding pocket of TRAF3, Y440 and Q442, abrogates these interactions [20]. Interestingly, a strong interaction was detected between FLAG-TRAF3 Y440/Q442A and Myc-p115 or EGFP-Sec16A (Figures 3C and 3D), implying that this interaction is independent of the TIM motif. Thus, it is not clear yet whether TRAF3 interacts directly with Sec16A or p115 or requires other components such as TFG ([46] and see Figure S1). Collectively, these data suggest that an intact TRAF3 molecule is required for its proper localization and interaction with components of the ER-to-Golgi vesicular pathway.

### Activation of intracellular RNA and DNA sensors leads to the formation of TRAF3-containing Golgi fragments

**Figure 4:**
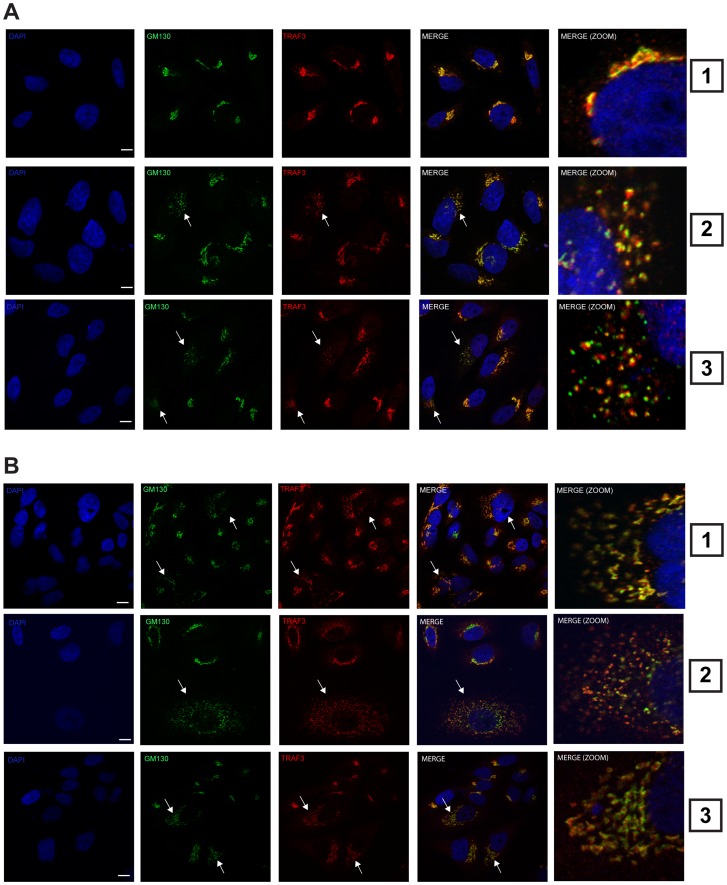
Activation of intracellular RNA and DNA sensors leads to the formation of TRAF3-contraining Golgi fragments. Confocal microscopy of HeLa cells stained for endogenous TRAF3, GM130, and the nucleus (DAPI) upon no treatment (**A**, panel 1), poly I:C treatment (**A**, panel 2) or poly dA:dT for 4 h (**A**, panel 3), infection with SeV (200 HAU/ml) (**B**, panel 1), RSV (MOI = 3) (**B**, panel 2), or Influenza A virus for 4 h (**B**, panel 3). Arrows indicate the relocalization and the colocalization of the Golgi apparatus with TRAF3 upon treatment. Bars represent 5 µm. One of three independent experiments with similar results is shown.

Our data demonstrate that TRAF3 does not associate with the mitochondrial network in resting cells (Figure 2A, panel 8). However, TRAF3 was demonstrated to link the mitochondrial membrane-bound protein MAVS to the activation of TBK1, which is required for IRF3/7 phosphorylation and type I IFN induction in response to viral infection [20], [47]. Therefore, we next addressed the subcellular localization of endogenous TRAF3 upon viral infection and RNA/DNA sensor pathway activation. Intracellular delivery of the double-stranded RNA mimicry molecule, poly I:C, or the dsDNA mimicry agent poly dA:dT resulted in disorganization of the ribbon-like structure of the Golgi apparatus, giving rise to the formation of Golgi ministacks containing GM130 (Figure 4A, arrows in panel 2 and 3). Importantly, the localization of endogenous TRAF3 followed these Golgi fragments. Similar observations were made in cells infected with RIG-I inducers, Sendai virus (SeV), Respiratory Syncytial Virus (RSV) and Influenza virus (Figure 4B).

**Figure 5:**
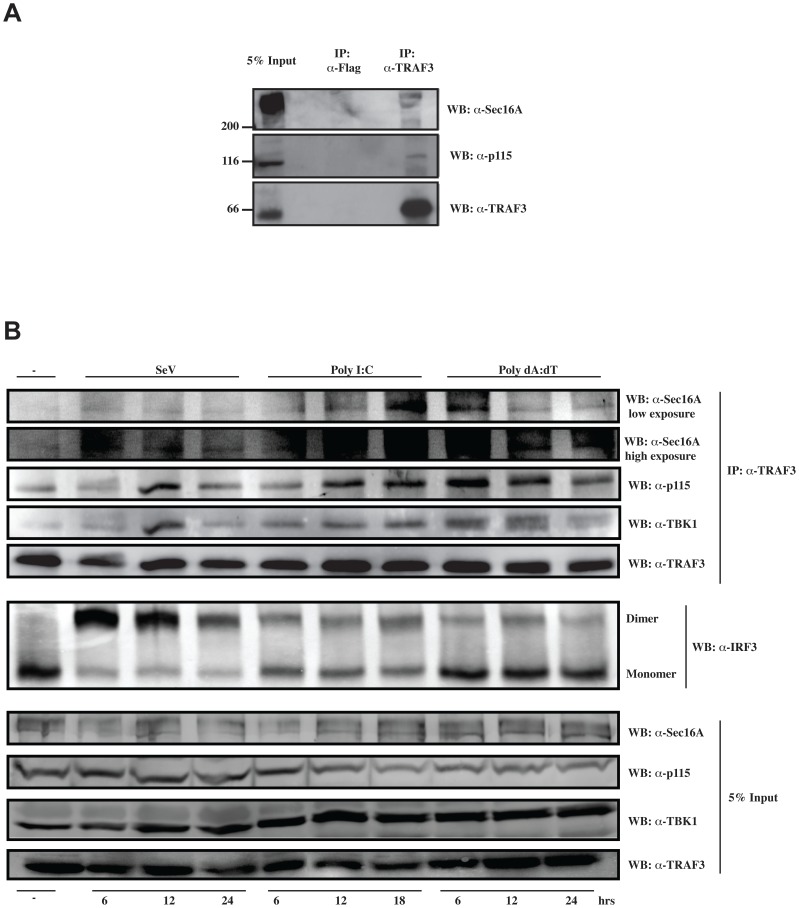
p115 and Sec16A associate with TRAF3 following cytosolic RNA and DNA sensor activation. (**A**) Whole-cell lysates (HeLa cells) were prepared and subjected to immunoprecipitation assays using TRAF3 (H-20) or isotype control antibodies followed by immunoblotting for the presence of p115, Sec16A, and TRAF3. (**B**) HeLa cells were treated as indicated for different periods of time. Whole-cell lysates were prepared and subjected to immunoprecipitation assays using TRAF3 (H-20) antibody followed by immunoblotting for the presence of p115, Sec16A, TBK1 and TRAF3. The Native-PAGE assay was conducted on the same cellular extracts to demonstrate the dimerization and activation of IRF-3 upon indicated treatments. One of three independent experiments with similar results is shown.

Additionally, we addressed the association of TRAF3 with p115- and Sec16A-containing complexes upon PAMP exposure. In unstimulated cells, a weak but constitutive association of endogenous TRAF3 with endogenous Sec16A and p115 was detected (Figure 5 A and B). However, upon viral infection or transfection with poly I:C or poly dA:dT, immunocomplexes containing endogenous TRAF3 were enriched with p115 and Sec16A. Importantly, the induced association of TBK1 with TRAF3 closely mirrored the presence of p115 and Sec16A. (Figure 5B). From these results we hypothesized that the localization of TRAF3 to the ER-to-Golgi compartment and the Golgi fragmentation of the latter into punctate structures might be required for the proper positioning of TRAF3 with MAVS.

### Sec16A and p115 are required for TRAF3 localization to the ER-to-Golgi transport compartment and the proper recruitment to MAVS

To verify this hypothesis, loss-of-function experiments were conducted using HeLa cells exposed to siRNA duplexes targeting Sec16A and p115. As previously observed for p115 and Sec16A [24], [25], [48], [49], reducing the expression level of Sec16A or p115 led to a drastic disorganisation of the Golgi paralleled by a relocalization of TRAF3 as observed by the formation of small punctate structures (Figure S4A, panels 2 and 4; Figure S4B, panel 2). However, the majority of these GM130 positive punctae do not colocalize with TRAF3 and thus appear to be different from those observed following dsRNA and dsDNA sensing (compare Figure S4A, panel 4 with Figure 4).

**Figure 6:**
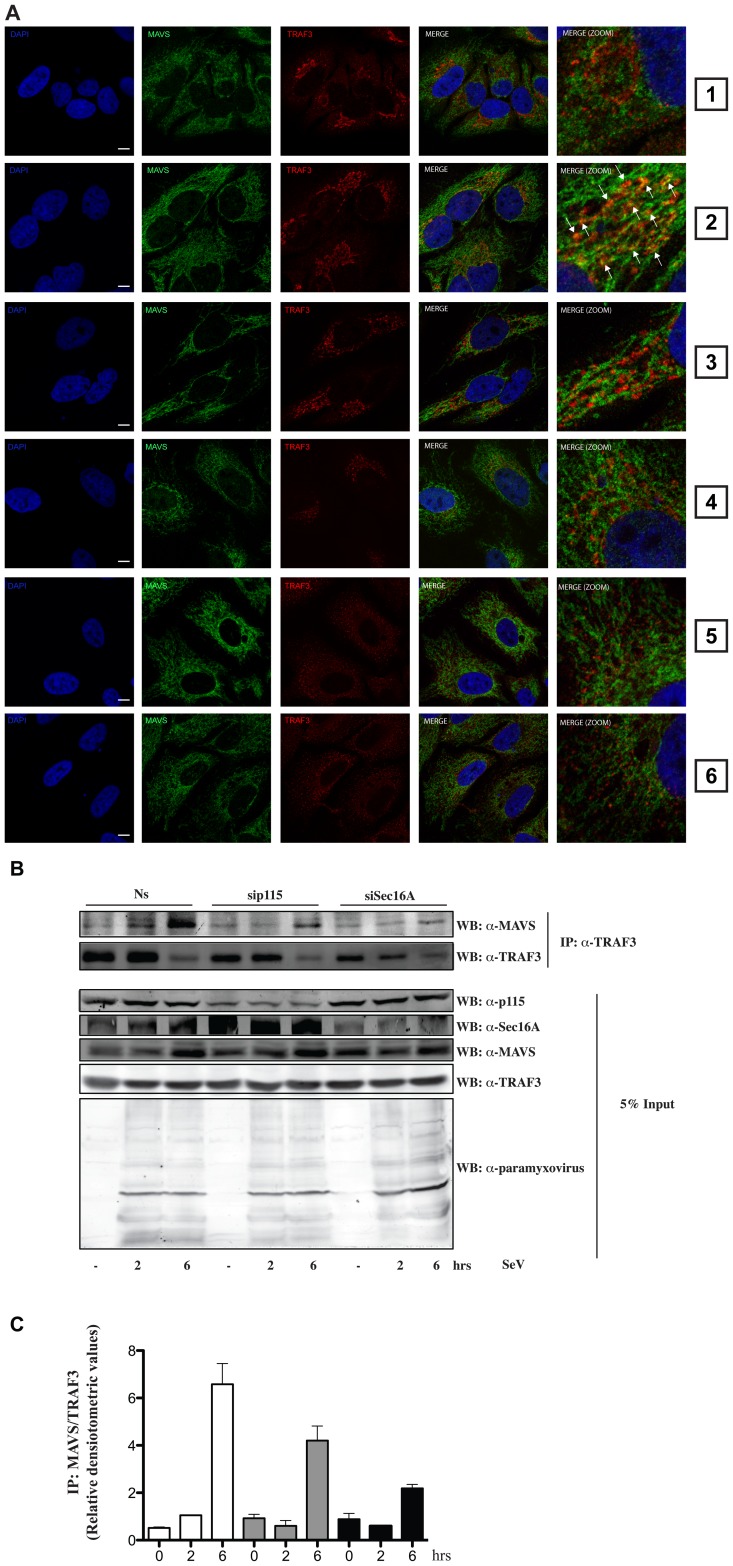
Sec16A and p115 are required for the proper positioning of TRAF3 along the mitochondrial network. (**A**) Confocal microscopy of HeLa cells transfected with 40 nM nonsilencing RNA duplexes (panels 1 and 2) or 40 nM siRNA duplexes that specifically target Sec16A (panels 3 and 4) or p115 (panels 5 and 6) and stained for MAVS and endogenous TRAF3 upon no treatment (panels 1, 3 and 5) or SeV infection (200 HAU/ml) for 4 h (panels 2, 4 and 6). Arrows indicate the colocalization of TRAF3 with MAVS. Bars represent 5 µm. One of three independent experiments with similar results is shown. (**B**) p115 and Sec16A were silenced in HeLa cells as described in (**A**) and infected with SeV for indicated periods of time. Whole-cell lysates were subjected to immunoprecipitation using an anti-TRAF3 (H-20) antibody followed by immunoblotting for the presence of MAVS and TRAF3. Immunoblot analysis against p115, Sec16A, TRAF3 and SeV proteins are also shown (Input). One of two independent experiments with similar results is shown. (**C**) Densitometric analysis of the binding activity of MAVS to TRAF3 presented in Figures 6B. Data represent the ratio of immunoprecipitated MAVS over immunoprecipitated TRAF3 and are means +/− S.D. of two experiments.

Next we examined the effect of reducing the expression level of Sec16A or p115 on the ability of TRAF3 to colocalize with MAVS upon SeV infection. As expected, TRAF3 localization reorganized into punctate structures following SeV infection, allowing a significant proportion of TRAF3 to colocalize with MAVS (Figure 6A, panel 2). This effect was severely compromised by reducing the expression of Sec16A or p115 (Figure 6A, compare panels 4 and 6 with panel 2). Additionally, co-immunoprecipitation experiments revealed that TRAF3 formed an immunocomplex with MAVS upon SeV infection. Interestingly, silencing the expression level of p115 or Sec16A clearly blunted the ability of TRAF3 to bind to MAVS upon SeV infection (Figures 6B and 6C). Thus loss-of-function experiments targeting p115 and Sec16A led to a mislocalization of TRAF3 and its subsequent incapacity to associate with MAVS upon RLH pathway activation.

**Figure 7:**
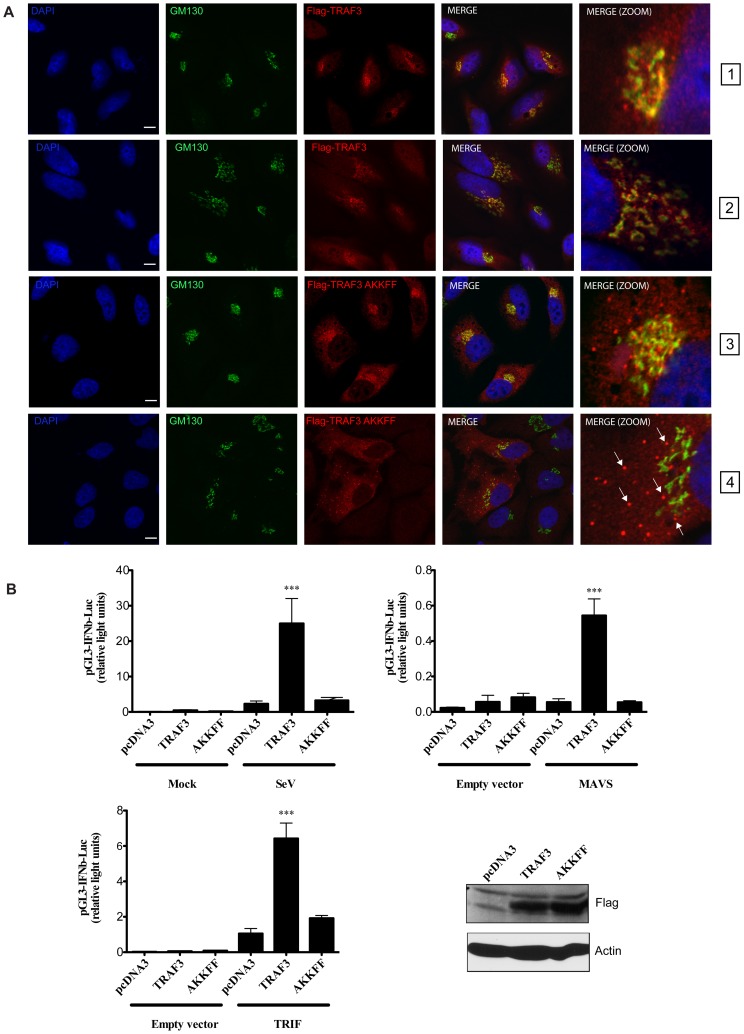
Enforced retention of TRAF3 at the ER-to-Golgi compartment negatively regulates type I IFN response. (**A**) Confocal microscopy analysis of FLAG-tag and GM130 performed in HeLa cells expressing FLAG-TRAF3 (panels 1 and 2) or FLAG-TRAF3-AKKFF (panels 3 and 4) upon no infection (panels 1 and 3) or SeV infection (200 HAU/ml) (panels 2 and 4) for 4 h. The nuclei were stained with DAPI. Arrows indicate TRAF3 aggregates. One of two independent experiments with similar results is shown. Bars represent 10 µm. (**B**) Hec1B cells were co-transfected with luciferase reporter plasmid pGL3-IFNβ (250 ng) and indicated plasmids (250 ng) for 24 h and infected with SeV (200 HAU/ml) for 16 h. Hec1B cells were also co-transfected with luciferase reporter plasmid pGL3-IFNβ (250 ng), empty vector or MAVS or TRIF (25 ng) along with indicated plasmids (250 ng) for 24 h. Relative luciferase activity was measured as described in Materials and Methods. Mean values +/− S.D. of triplicate determinations are shown (*** P<0.001). One of four independent experiments with similar results is shown. Cellular extracts from transfected Hec1B cells were also prepared and subjected to immunoblot analysis using indicated antibodies (right lower panel).

This prompted us to ask whether enforced retention of TRAF3 at the ER-to-Golgi compartment could negatively influence the type I IFN response. In order to verify this, a TRAF3 mutant containing a COPI and COPII sorting signal peptide [50], namely “AKKFF” [51], at its C-terminal end was generated and used in confocal microscopy and reporter gene assays. Confocal microscopy experiments revealed that addition of dilysine and dihydrophobic residues to the C-terminal end of TRAF3 resulted in the formation of large TRAF3 aggregates which failed to colocalize with the Golgi marker GM130 upon infection with SeV (Figure 7A). Consequently, the ability of the TRAF3-AKKFF mutant to mediate TRAF3-dependent synergistic activation of the IFNβ promoter was drastically reduced (Figure 7B), which is likely due to less binding to MAVS (Figure S5).

Altogether, these results indicate that the localization of TRAF3 to the ER-to-Golgi compartment is involved in the proper positioning of TRAF3 within the mitochondrial network and the induction of type I IFN innate immune response.

### Sec16A and p115 influence the type I IFN antiviral response at the transcriptional level

**Figure 8:**
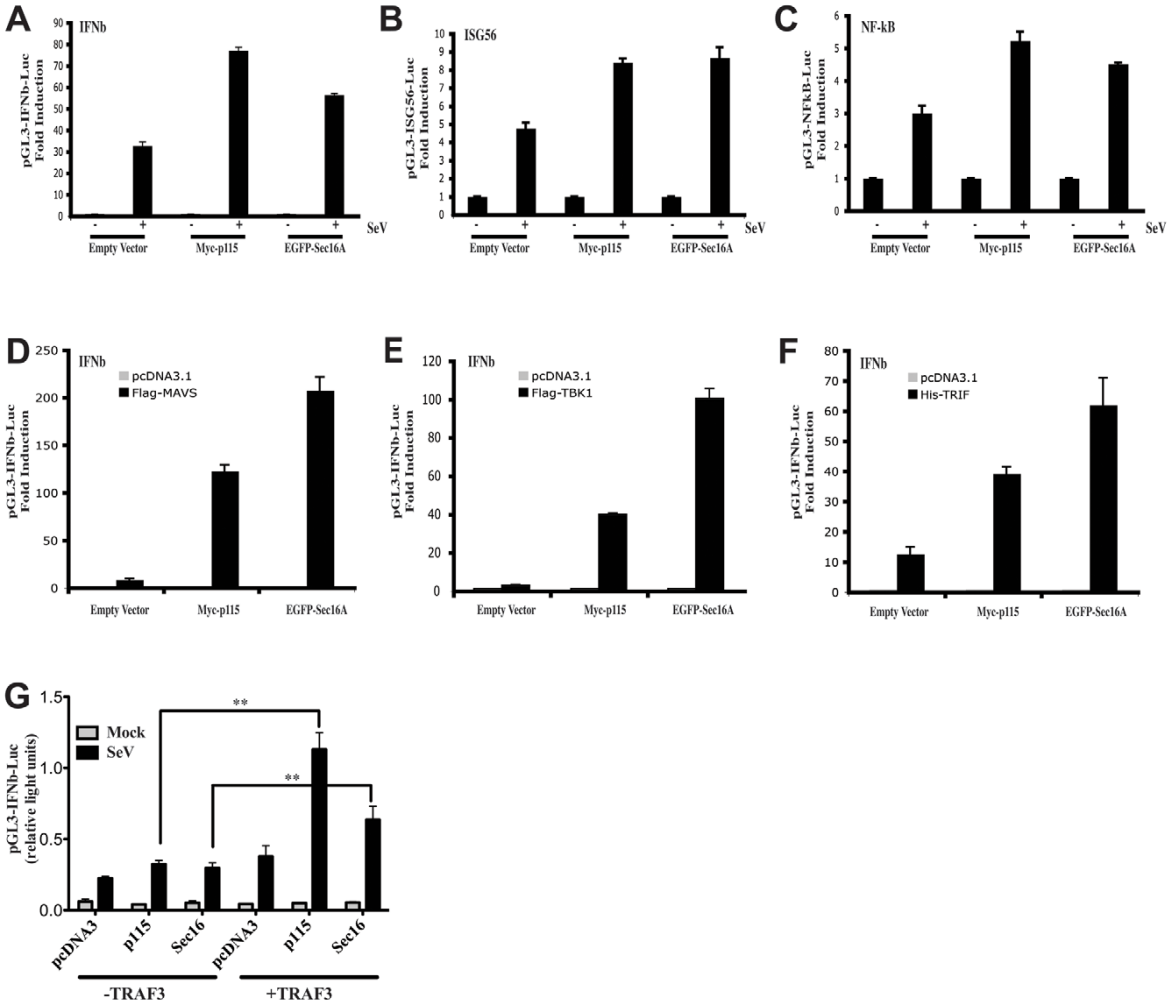
Sec16A and p115 influence the type I IFN antiviral response at the transcriptional level. (**A–C**) Hec1B cells were co-transfected with the indicated luciferase reporter genes together with 125 ng of empty vector, Myc-p115 or EGFP-Sec16A. Data are expressed as fold-induction following SeV infection (16 h) over the corresponding non-infected condition. (**D–F**) Hec1B cells were co-transfected with pGL3-IFNβ-luciferase reporter gene together with 125 ng of empty vector, Myc-p115 or Sec16A and 15 ng of FLAG-MAVS (**D**), 100 ng of FLAG-TBK1 (**E**), or 15 ng of His-TRIF (**F**). Data represent the fold-activation over the corresponding vector control. Each value represents the mean +/− S.D. of triplicate determinations. The data are representative of at least four different experiments with similar results. (**G**) TRAF3 knockout MEF cells were co-transfected with 250 ng of luciferase reporter plasmid pGL3-IFNβ, 500 ng of pcDNA3 or FLAG-TRAF3 plasmid and 375 ng of indicated plasmids. At 24 h post-transfection, cells were left uninfected or infected with SeV (200 HAU/ml) for 16 h and relative luciferase activity was measured as described in Materials and Methods. Mean values +/− S.D. of triplicate determinations are shown (** P<0.01). One of three independent experiments with similar results is shown.

The results presented above suggest a role for the ER-to-Golgi compartment in TRAF3-dependent innate immune response. To investigate whether Sec16A or p115 play a role in the type I IFN response, we overexpressed both proteins in Hec1B cells and assessed NF-κB and IRF-3 transcription factor activation using reporter gene assays. Without any stimulation, overexpression of either protein did not significantly activate the IFNβ promoter. However, following viral infection, the response was increased in cells overexpressing Sec16A or p115 (Figure 8A). Overexpression of Sec16A and p115 also increased the activation of the ISG56 promoter (IRF3-dependent promoter) (Figure 8B) and the NF-κB-dependent promoter (Figure 8C) following SeV infection. Moreover, we observed a synergistic effect on IFNβ promoter activity when Sec16A or p115 were co-expressed with MAVS (Figure 8D), TBK1 (Figure 8E) and, interestingly, the TLR3 essential effector TRIF (Figure 8F). Similar results were also obtained for the ISG56 promoter and the NF-κB-dependent promoter (Figure S6). To further substantiate that the positive transcriptional effect of Sec16A and p115 is dependent on TRAF3, TRAF3-knockout MEF cells were transfected with p115 and Sec16A in the presence or absence of TRAF3 and used in the IFNβ promoter reporter assay. As suspected, the enhanced promoter activation, induced by ectopically expressed p115 and Sec16A, was entirely dependent on the presence of TRAF3 (Figure 8G). Thus, when expressed in relatively low amounts in Hec1B and MEF cells (not shown), p115 and Sec16A positively participate in a TRAF3-dependent type I IFN response, probably reflecting the ability of a subpopulation of cytoplasmic TRAF3 to further associate with the ER-to-Golgi components under these conditions of mild ectopic expression.

Interestingly, several recent studies have demonstrated that overexpression of p115 or Sec16A in highly transfectable cell lines and depletion of Sec16 or p115 resulted in identical cellular outcomes (i.e. Golgi fragmentation (see Figure S4 and Figure 6) and delayed ER-to-Golgi transport), thereby suggesting that they are required in stoichiometric amounts [24], [25], [52]. Thus, when ectopically expressed in high amounts in 293T cells, p115 and Sec16A were expected to blunt TRAF3-dependent transcriptional activation. Indeed, transfection of increasing amounts of p115 or Sec16A efficiently blunted TRIF-, RIG-I-, and MAVS-induced IFNβ promoter activation (Figure S7A–C) as well as NF-κB promoter activation (data not shown). Importantly, adding increasing amounts of TRAF3 in this specific reporter gene assay dose-dependently reversed the inhibitory effect of p115 and Sec16A, once more substantiating the relationship that exist between Sec16A, p115 and TRAF3 (Figure S7D). Moreover, transfection of these plasmids also blunted TBK1-induced ISRE promoter activation (Figure S7E), but did not affect the transactivation response induced by the use of a constitutively active form of IRF-3 (IRF3-5D) (Figure S7F), suggesting that the ER-to-Golgi compartment plays upstream of IRF-3 in type I IFN signalling.

**Figure 9:**
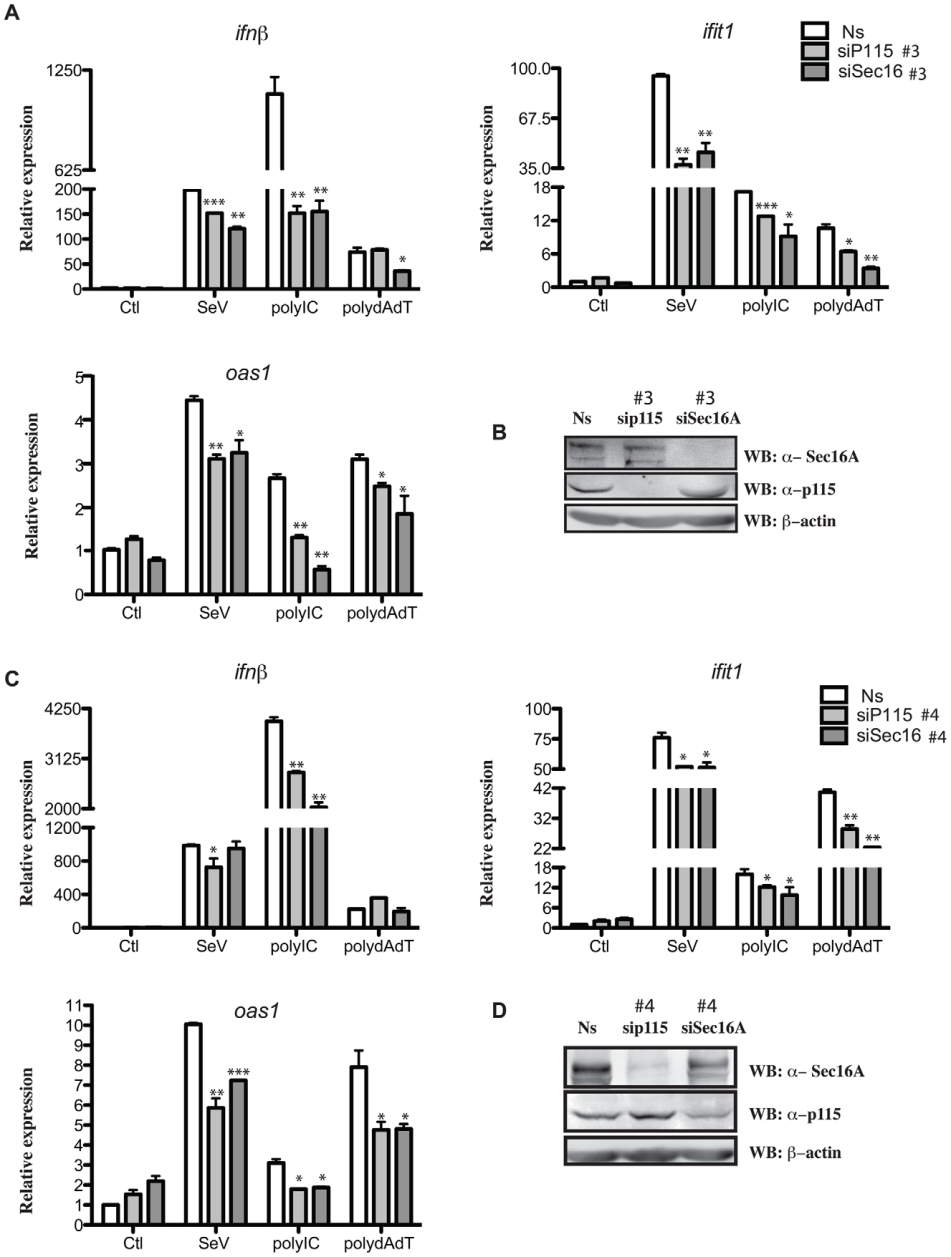
Requirement of Sec16A and p115 for optimal type I IFN innate immune response in cells exposed to cytosolic DNA and RNA sensor ligands. (**A–D**) HeLa cells were transfected with nonsilencing (Ns) RNA duplexes or two different sets of siRNA duplexes that specifically target p115 or Sec16A as indicated. 72 h post-transfection, cells were left untreated (Ctl) or stimulated with poly I:C (2.5 µg/ml), poly dA:dT (1 µg/ml) or SeV (200 HAU/ml) for 6 h to 8 h. RNA was extracted and analyzed by RT-qPCR using primers for *ifnβ*, *ifit1*, *oas1*. Data are means +/− S.D. (n = 3). * Significantly below the induction response; * P<0.05, ** P<0.01, *** P<0.001. (**B and D**) Cellular extracts were also prepared and subjected to immunoblot analysis using indicated antibodies. One of three independent experiments with similar results is shown.

**Figure 10:**
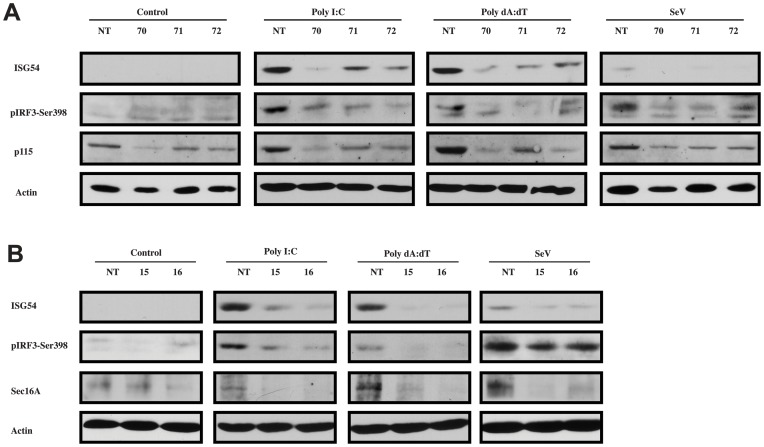
p115 and Sec16A are required for optimal IRF-3 activation in response to activation of cytosolic RNA and DNA sensors. HeLa cells were infected with lentiviral vectors encoding shRNA targeting p115 (**A**) or Sec16A (**B**) and non-targeting (NT) control shRNA for 24 h followed by puromycin selection (1.5 µg/ml) for 5 days. Cells were left untreated or stimulated with poly I:C (1 µg/ml), poly dA:dT (1 µg/ml) or SeV (200 HAU/ml) for 16 h. Whole-cell lysates were prepared and subjected to immunoblot analysis with indicated antibodies. One of two independent experiments with similar results is shown.

To further confirm the implication of p115 and Sec16A in the type I IFN response, loss-of-function experiments were conducted next. As suspected, an RNAi approach targeting Sec16A and p115, which leads to Golgi fragmentation (see Figure S4 and Figure 6) significantly diminished *Ifnb*, *ifit1* (ISG56), and *oas1* mRNA induction following poly I:C and poly dA:dT transfection and SeV infection (Figure 9). To verify whether this approach affected IRF-3 activation and the induction of an IRF-3-dependent antiviral protein [53], we next verified the phosphorylation state of IRF-3 and the induction of ISG54 in HeLa cells expressing either shRNA duplexes targeting p115 and Sec16A or cells expressing a non-targeting (NT) shRNA duplex. The phosphorylation of IRF-3 and the expression of ISG54 were readily observed upon SeV infection, poly I:C and poly dA:dT transfection in HeLa cells expressing the NT shRNA duplex but was clearly reduced in cells expressing different shRNA duplexes targeting p115 (Figure 10A) and Sec16A (Figure 10B). Altogether, these data indicate that TRAF3 localization to the ER-to-Golgi vesicular pathway is necessary for a proper type I IFN response.

## Discussion

Gene disruption strategies have revealed that TRAF3 plays a major role in the type I IFN response [12], [13]. However, how TRAF3 assembles into functional signalling complexes is still not fully understood. In general, TRAF3 is thought to reside in the cytosol and translocate to surface membrane receptors upon engagement of CD40 or other TNFR family members [54]. Akin to its role in MyD88-dependent cytokine production and TRIF-dependent type I IFN production [55], TRAF3 conceivably also has the capacity to associate with endosomal compartments enriched in TLR3, TLR4, TLR7, TLR8 and TLR9 receptors [56]. Additionally, upon RLH activation TRAF3 interacts with MAVS and TRADD to trigger IRF-3 phosphorylation through the adaptor molecule TANK and the IKK-related kinases TBK1 and IKKi [20], [21]. However, how TRAF3 associates with MAVS upon RLH activation remains unanswered.

**Figure 11:**
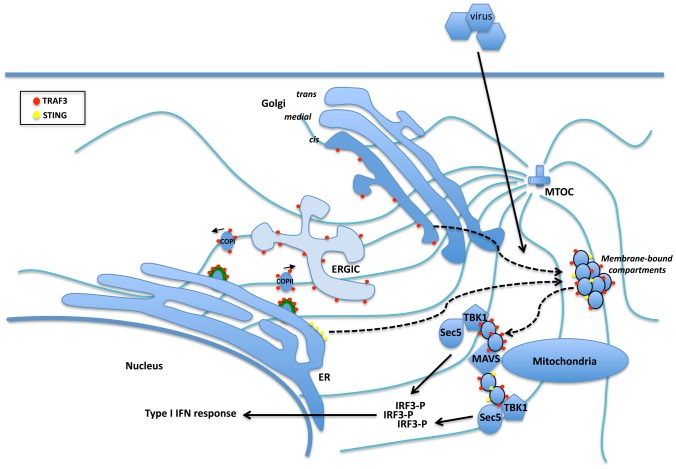
Through its ability to interact and colocalize with components of the ERES (Sec16A, depicted as thick green lines), ERGIC (ERGIC53 and p115) and the cis-Golgi apparatus (p115 and GM130), a subpopulation of TRAF3 (red circles) resides in the ER-to-Golgi vesicular compartment in non-infected cells. Upon dsRNA and dsDNA sensing, the cis-Golgi disorganizes into punctate structures, giving rise to membrane-bound compartments composed of at least GM130 and TRAF3 (dashed line). We propose that these membrane-bound compartments allow the proper positioning of TRAF3 with MAVS at Mitochondrial-Associated endoplasmic reticulum Membranes (MAM) [86]. There, being in close proximity with a component of the exocyst (sec5) and the translocon (Sec61β), TRAF3 allows the activation of TBK1 and IRF3 leading to activation of the type I IFN response. A similar scenario was recently proposed for STING (yellow circles) where in response to DNA virus infection, it traffics from the ER to the cis-Golgi apparatus and finally to a distinct perinuclear region for the activation of TBK1 [61], [62]. MTOC: microtubule-organizing center.

Herein, we report that TRAF3 localizes to the ER-to-Golgi compartments through its ability to interact with p115- and Sec16A-containing complexes. A pharmacological approach using the microtubule depolarizing agent nocodazole led to the redistribution of TRAF3 into small punctate cytoplasmic structures discrete from the ER along with both Golgi matrix proteins p115 and GM130. Both the structure and positioning of the Golgi apparatus have been shown to be highly dependent on the microtubule cytoskeleton [57]. Interestingly, a link between TRAF3 and the microtubule network has been already established in a previous study through its interaction with Microtubule-Interacting Protein that associates with TRAF3 (MIP-T3) [58]. TRAF3 was dissociated from this complex upon CD40L stimulation and, consequently, it was suggested that microtubule association of TRAF3 could be responsible for directing TRAF3 to defined membrane microdomains in the cell. A similar scenario is proposed here where, in response to viral infection, the association of TRAF3 with complexes containing p115 and Sec16A at the ER-to-Golgi vesicular pathway may play an important role in positioning TRAF3 with MAVS (see Figure 11). Indeed, the following findings suggests a role for Sec16A and p115 in the TRAF3-mediated RLH type I IFN response: (1) Sec16A and p115 are found in immunocomplexes containing TRAF3, but not TRAF2 or TRAF6; (2) inactivation of TRAF3 by deletion of its N-terminal RING finger domain and the C-terminal TRAF domain displaces TRAF3 from the ER-Golgi transport compartments; (3) in non-treated cells, TRAF3 colocalizes and tightly associates with p115, Sec16A, ERGIC53 and GM130, markers of the ER-to-Golgi vesicular compartment; (4) activation of the RLH pathway leads to reorganization of the Golgi apparatus into punctate structures containing TRAF3 and GM130; (5) an increased association between TRAF3, Sec16A, p115 and TBK1 is observed in virally-infected, dsRNA- and dsDNA-transfected cells; (6) mild overexpression of both proteins enhances SeV-, TBK1- and MAVS-stimulated IFNβ, ISG56 and NF-κB promoter induction; (7) knocking down the expression level of p115 or Sec16A affects the cellular distribution of TRAF3, impairs its capacity to associate with MAVS and diminishes the type I IFN response following poly I:C or polydA:dT transfection and SeV infection; and (8) enforced retention of TRAF3 at the ER-to-Golgi compartment by the addition of a COPI and COP II sorting signal peptide impairs TRAF3 recruitment to the cis-Golgi and diminishes the type I IFN response. Thus, we propose that these two trafficking proteins, Sec16A and p115, form a complex with TRAF3 at ER-to-Golgi transport compartments in order to ensure its proper recruitment to the mitochondrial network during a viral infection. Interestingly, enforced expression of Sec16A or p115 also increases TRIF-mediated IFNβ promoter activation, reinforcing the role for the ER-to-Golgi vesicular compartment in TLR3 and TLR4 signalling, as recently reviewed [59].

In support of our findings, the ER-to-Golgi transport compartment seems to also host several proteins involved in type I IFN signalling such as TRADD [21], the translocon [31] and potentially the exocyst [32] (Clement and Servant, unpublished observations). How these proteins cooperate with TRAF3 at the ER-to-Golgi transport compartments is currently unclear and will be the objective of future studies. Nevertheless, all these data suggest a model where vesicles and/or membranes originating from reorganized ER-to-Golgi compartments come in close proximity with the mitochondrial network in order to facilitate the assembly of a functional MAVS signalling complex.

In addition to its role in the RNA sensing pathways, STING is now considered an important effector of innate immune signalling in response to DNA pathogens [60]. Interestingly, STING is an ER-resident protein, which in response to dsDNA treatment, was recently demonstrated to traffic from the ER to the Golgi [61], [62] giving rise to punctate structure formation [62]. It is likely that the use of dsDNA (polydA:dT) used in our study might activate both the RNA-dependent pathway (through RNA polymerase III [63]) and the recently described DNA-dependent pathway (through IFI16 [2]), allowing TRAF3-loaded punctae to interact with both MAVS and STING respectively for proper innate immune signalling (Figure 11). Even though this needs to be investigated further, we speculate that the membranous network composed of the ER, Golgi and mitochondria provides a convenient platform on which antiviral cell-signalling complexes are arranged and optimally activated.

It is noteworthy that, as a common feature, plus-stranded RNA viruses have the ability to induce cytoplasmic membrane rearrangements that facilitate their replication. Consequently, the formation of these RNA replication complexes results in dramatic reorganization of the secretory pathway of host cells [64]. For example, poliovirus-infected cells accumulate membranous vesicles derived from COPII vesicles [65] whereas Kunjin virus induces “convoluted membranes” that contain markers from the ERGIC [66]. The precise role for this internal membrane rearrangement in the virus propagation and virus-host interaction requires further investigation. Nevertheless, localization of TRAF3 and TRADD to these vesicular transport compartments could represent a cellular strategy to increase the rate of RNA detection and the formation of an effective signalling complex at the mitochondrial membrane. The observation that TRADD translocates from the cytoplasm to the mitochondria during Influenza A virus infection supports this model [67]. Additionally, recent observations highlight the fact that viruses have evolved a variety of mechanisms involving the Golgi apparatus to specifically block TRAF3 recruitment into a functional signalling complex. Notably, the SARS Coronavirus M protein, a Golgi localized protein, was recently found to impede the formation of a TRAF3-TANK-TBK1/IKKi complex at the Golgi apparatus [68]. The NY-1 strain Hantavirus glycoprotein (Gn) was also shown to disrupt TRAF3-TBK1 interaction by interacting with TRAF3 through its cytoplasmic tail [69].

The notion of cellular proximity to favor exchanges and signalling events between organelles has been an intense field of interest for many years. Recently, mitofusin 2 present on the ER was shown to tether the ER to mitochondria in order to promote efficient Ca^2+^ uptake into the mitochondria for oxidative phosphorylation purposes. Interestingly, mitofusin 2 was also shown to inhibit RLH pathway signalling by interacting with the C-terminal of MAVS [70]. Furthermore, the Golgi localization of the glycolipid GD3 is important for its transport to the mitochondria after TNF-α stimulation [71], [72]. Membrane scrambling between Golgi and mitochondria following Fas stimulation is another example pointing to the connection between different cellular organelles [73]. Moreover, signalling at the Golgi apparatus and endosomes has been observed for different types of membrane-bound receptors [56], [74] and protein kinase cascades [75].

Although Bouwmeester and colleagues reported an NF-κB-inducing kinase-dependent interaction between Sec16A and NF-κB 2/p100 in an exhaustive study mapping the human TNF-α/NF-κB signal transduction network [76], a role for the ER-to-Golgi vesicular pathway in RLH-induced innate immune response was still unknown until now. Future characterization of the TRAF3 interactome will undoubtedly help to understand the molecular relevance of the specific subcellular localization of TRAF3 for an optimal type I IFN response.

## Materials and Methods

### Reagents, antibodies and plasmids

Commercial anti-GM130 antibody was purchased from BD Transduction (San Jose, CA). The monoclonal anti-FLAG epitope (M2), the polyclonal anti-FLAG and the anti-β-actin (clone AC-74) were obtained from Sigma (Oakville, Ontario, Canada). The c-Myc (9E10) monoclonal antibodies, as well as the polyclonal p115 (H-300) and TRAF3 (C-20, H-20, and G-6) antibodies were purchased from Santa Cruz (Santa Cruz, CA). The anti-GFP (monoclonal 1218) antibody and the polyclonal goat anti-GFP antibody were obtained from ABCAM (Cambridge, MA) and US Biological (Swampscott, MA) respectively. The anti-ERGIC53 and anti-calnexin antibodies were from Enzo Life Sciences, anti-p-IRF3 Ser398 was from Millipore (Billerica, MA) and anti-ISG54 was from Novus Biologicals (Littleton, CO). The polyclonal anti-Sec16A and p115 antibodies were obtained from Bethyl Laboratories and Santa Cruz. The plasmid encoding for EGFP-Sec16A was a kind gift of Dr. David Stephens (University of Bristol, UK). Human TRAF3 and p115 cDNAs were amplified from the MGC bank collection and respectively subcloned in pcDNA3 and pTag2B (FLAG) or pTag3B (Myc) vectors (Invitrogen, Burlington, ON, Canada). Human TRAF6 cDNAs were purchased from Origene (Rockville, MD) and subcloned in the pTag2B/3B vectors. The pFLAG-CMV2-TBK1 and pFLAG-TRAF3 Y440/Q442A were gifts from Drs. John Hiscott (McGill University). pcDNA3.1-FLAG-MAVS construct was from Rongtuan Lin (McGill University). The pcDNA3-His-TRIF construct was from Dr. Daniel Lamarre (Université de Montréal). The pRK5-TRAF2-FLAG was obtained from Dr. Nathalie Grandvaux (Université de Montréal). pFLAG-CMV2 TRAF3 deletion mutants (1–117, 1–381, 114–568, 259–568 and 389–568) were from Dr. Carl Ware (La Jolla Institute for Allergy and Immunology). The IFNß reporter plasmid, pGL3-IFN-ß-LUC was described previously [77] as well as the ISG56-luciferase [78] and the NF-κB p2(2)TK reporter plasmids [77]. The pFLAG-TRAF3 mutant with C-terminal retention motif AKKFF was generated by PCR and subcloned in pCDNA3.1 (+) and pMRX-ires-puro (a kind gift from Dr. Shoji Yamaoka, Tokyo Medical and Dental University, Japan). Poly I:C was purchased from GE HealthCare (Waukesha, WI) and transfected with Lipofectamine2000 (Invitrogen) at final concentrations of 1.0 to 2.5 µg/ml. Poly dA:dT was from InvivoGen and used at 1 µg/ml. BFA and nocodazole were obtained from Calbiochem and used at a final concentration of 5 µg/ml.

### Cell culture, transfection and infections

HeLa, Hec1B, HEK 293, HEK 293T, HEK 293 QBI cell lines and TRAF3 knockout MEF cells (a kind gift from Dr. John Hiscott, McGill University) were maintained in Dulbecco's modified Eagle Medium supplemented with 10% fetal bovine serum. All DNA transfections in human cell lines were performed with Lipofectamine 2000 (Invitrogen) according to the manufacturer's protocol. Transient transfection of immortalized MEF cells was performed by microporation with the Microporator Apparatus (Montreal Biotech) according to the manufacturer's instructions. Sendai virus (SeV) was obtained from Specific Pathogen-Free Avian Supply (North Franklin, CT) and used at 200 HAU/ml. Respiratory Syncytial Virus (RSV.A2) (a kind gift from Nathalie Grandvaux, Université de Montréal) was used at a MOI of 3. Influenza A (PR8) virus was a kind gift from Dr. Rongtuan Lin (McGill University).

### Immunoblot analysis and immunoprecipitation

Preparation of whole cell extracts, co-immunoprecipitation studies, Native-PAGE and immunoblot analysis were performed as described previously [79]. A RIPA buffer (50 mM Tris-HCl, pH 7.4, 100 mM NaCl, 5 mM EDTA, 50 mM sodium fluoride, 40 mM β-glycerophosphate, 1 mM sodium orthovanadate, 1% Triton X-100, 0.1% SDS, 0.5% sodium deoxycholate, and protease inhibitors mixture (Sigma)) was used for the extraction of the TRAF3 AKKFF mutant. Antibodies were used as recommended by the manufacturers.

### Confocal microscopy

For immunofluorescence, cells were fixed with 4% paraformaldehyde (PFA) in PBS for 20 min followed by permeabilization with 0.1% Triton X-100 for 5 min. Cells were washed with PBS (pH 7.2) and blocked with 0.5% BSA in PBS. Anti-FLAG antibody (M2, Sigma) was used at 1∶1000, anti-GM130; 1∶100, anti-ERGIC53; 1∶100, anti-FLAG polyclonal antibody; 1∶400, anti-GFP (ABCAM); 1∶100, anti-Myc 9E10; 1∶100, anti-TRAF3; 1∶200, anti-P115; 1∶100, anti-Sec16A; 1∶200, and anti-MAVS; 1∶100. Secondary fluorophore-conjugated antiserum (Alexa Fluor 488 and 564) was obtained from Molecular Probes (Eugene, OR) and used at 1∶500 in PBS 0.5% BSA. The nucleus was revealed by 4′,6-diamidino-2-phenylindole (DAPI) staining. The confocal micrographs represent a single optical section through the plane of the cell. Images were acquired with LSM v3.2 software (Zeiss) on a LSM 510 inverted microscope (Zeiss, Germany) with a plan-apochromat 63×/1.4 oil disc lens using 405 nm in conjunction with a LP 505 for DAPI, 488 nm in conjunction with a BP 505–530 for Alexa 488, and 543 nm in conjunction with BP 560–615 for Alexa 568. Images were assembled in Adobe Photoshop CS 3.0.

### FLAG affinity purification and mass spectrometric analysis

FLAG-affinity purification was performed as described previously [80] with the following modifications. Detergent concentration in the lysis buffer was 0.5% NP-40; the lysis buffer was added at 4 ml/g wet cell pellet, and cells were subjected to passive lysis (30 minutes) followed by one freeze-thaw cycle and centrifugation. Immunoprecipitation was performed on the cleared lysate by adding 25 µl packed FLAG M2 beads (Sigma) and incubating for two hours. Beads were washed three times in lysis buffer, and three times in 50 mM ammonium bicarbonate. Samples were eluted with ammonium hydroxide, lyophilized in a speed-vac, resuspended in 50 mM ammonium bicarbonate (pH 8–8.3), and incubated at 37°C with trypsin overnight. The ammonium bicarbonate was evaporated, and the samples were resuspended in HPLC buffer A2 (2% acetonitrile, 0.1% formic acid), then directly loaded onto capillary columns packed in-house with Magic 5 µm, 100A, C18AQ. MS/MS data was acquired in data-dependent mode (over a 2 hr acetonitrile 2–40% gradient) on a ThermoFinnigan LTQ equipped with a Proxeon NanoSource and an Agilent 1100 capillary pump. Acquired RAW files were converted to mgf format using ProteoWizard. The searched database was human RefSeq (version 45). *.mgf files were searched with the Mascot search engine (version 2.3) using the following variable parameters: semi trypsin digestion, one missed cleavage allowed, asparagine deamidation and methionine oxidation. The fragment mass tolerance was 0.6 Da (monoisotopic mass), and the mass window for the precursor was +/−3 Da (only +2 and +3 charge ions were processed). Mascot results were parsed for further analysis into a LIMS system developed at the Samuel Lunenfeld Research Institute [81]. Scoring of specific interactors for FLAG-TRAF3 and FLAG-p115 was performed using the statistical tool SAINT (Significance Analysis of INTeracome). SAINT converts label free quantification, such as spectral counts, for each prey protein identified in a purification of a bait into the probability of true interaction between the two proteins [82], [83]. SAINT can calculate a probability of interaction even for proteins proteins frequently detected in AP-MS experiments, providing that a quantitative enrichment is detected in the purification of the sample [84]. For each bait, two biological replicates were used. Twelve negative control runs (consisting of cells expressing the FLAG tag alone) were processed in parallel and combined into 5 virtual controls for SAINT modeling. SAINT calculates scores differently depending on the availability of negative control purifications, and thus the implementation for spectral count data incorporating control purification data was used (details are described in [82]). The probability score was first computed for each prey in independent biological replicates separately (iProb). Then the final probability score for a pair of bait and prey proteins was calculated by taking the average of the probabilities in individual replicates (AvgP); final results with AvgP≥0.5 were further inspected. A manual cross-reference against a database containing >1000 independent FLAG AP-MS runs was finally performed to identify potential proteins frequently detected in AP-MS experiments and were removed from the final dataset.

### RNA interference

HeLa cells were transfected with 40 nM siRNA using Lipofectamine2000 (Invitrogen). siRNA p115, Sec16A and the non-targeting pool siRNA duplexes were purchased from Dharmacon (Lafayette, CO). Sequences are as follow: Sec16A (#3: 5′-ggagagcuuucgcgcugua-3′; #4: 5′-ccucaguccucuagcgugu-3′) and p115 (#3: 5′-guuauuauguggagguuug-3′; #4: 5′-ugauggagguauaguaguu-3′). shRNA vectors targeting p115 (TRCN0000065070, TRCN0000065071, TRCN0000065072) and Sec16A (TRCN0000246015, TRCN0000246016) and non-Targeting control shRNA were purchased form Sigma (St. Louis, MO). Lentiviral vector production and transduction was conducted as described previously [85].

### RNA isolation and qPCR analysis

After stimulation, total RNA was extracted from HeLa cells using Trizol reagent (Invitrogen). 2 µg of RNA was reverse transcribed using the High Capacity cDNA Reverse Transcription Kit with random primers (Applied Biosystems) as described by the manufacturer. SYBR green PCR reactions were performed using 2 µl of cDNA samples (25–50 ng), 5 µl of the Fast SYBR qPCR Master Mix (Applied Biosystems) and 10 pmol of each primer in a total volume of 10 µl. The IFN qRT-Primer set for real-time quantification of the IFN response (IFNβ, ISG56 (*ifit1*) and OAS1) was purchased from InvivoGen (San Diego, CA). The ABI PRISM 7900HT Sequence Detection System (Applied Biosystems) was used to measure the amplification level. All reactions were run in triplicate and the average Cts were used for quantification. TBP (TATA binding protein) was used as endogenous control.

### Reporter gene assays

Subconfluent Hec1B, HEK 293 QBI and 293T cells in 24 well-plates were transfected with 25 ng of pRL-TK reporter (renilla luciferase for internal control) and 125 ng of pGL3-IFN-β-LUC, pGL3-ISG56-LUC, pGL3-ISRE or pGL3-NF-κB-LUC using the conventional CaPO_4_ transfection protocol (for Hec1B, HEK 293 QBI cells and 293T cells) or Lipofectamine 2000 (for TRAF3 knockout MEF cells). Cells were harvested 24 h post-transfection, lysed in passive lysis buffer (Promega, Madison, WI), and assayed for dual-luciferase activity using 10 µl of lysate according to the manufacturer's instructions. All firefly luciferase values were normalized to renilla luciferase to control for transfection efficiency.

### Statistical analysis

Statistical analyses were performed using GraphPad Prism version 5.0 for Mac (GraphPad Software, San Diego, CA). Comparison of two groups was carried out using a two-tailed unpaired *t*-test, and comparison of more than two groups was carried out with one-way ANOVA and a Bonferroni posttest. Statistical significance was accepted at a *P*-value below 0.05.

### ID numbers for genes

TRAF2; 7186, TRAF3; 7187, TRAF6; 7189, Sec16A; 9919, p115; 8615, MAVS; 57506, STING; 340061, ISG56; 3434, ISG54; 3433, TRIF; 148022, TBK1; 29110, RIG-I; 23586, MDA5; 64135, LGP2; 79132, AIM2; 9447, IFI16; 3428, IRF3; 3661, NIK; 9020, TRADD; 8717, TANK; 10010, IKKi; 9641, GM130; 2801, RIP1; 8737, IFNβ: 3456, NFκB: 4790, OAS1; 4938, ERGIC53; 3998, calnexin; 821, Sec61β; 10952; Sec5; 55770.

## Supporting Information

Figure S1 The TRAF3 interactome network. (**A**) AP-MS data with ≥0.5 AvgP SAINT value. Indicated baits and prey (HUGO gene names; USO1 is the gene name for p115) are listed, alongside the accession number (protein NCBI gi) for the prey, and SAINT output data. Columns are as follows («|» is a delimiter for biological replicates): «IP» are unique identifiers for the experiment in the ProHits database; «Spec» are the spectral counts in each individual experiment; «SpecSum» is the sum of the spectral counts across all analyses. «iProb» is the initial probability in an individual experiment; «crtlCounts» are the spectral counts in five virtual controls, as defined in Methods; «AvgP» is the average of the individual probabilities. The following proteins passed the SAINT threshold filter but were excluded from further analysis, based on high-frequency of detection in FLAG AP/MS analysis from HEK293 cells: EWSR1, FUS, HNRNPC, TUBB2C, HSPA9, HNRNPM, HNRNPH1, ABCA13 and CEP290. (**B**) Overlay of the filtered mass spectrometry data with literature-curated interactions (as reported in BioGRID version 3.1.76*). Data is visualized in Cytoscape**. The blue colored edges are from the mass spectrometry data in Figure S1A; the grey from literature-curated interactions. The thickness of the blue edges corresponds to the number of spectral counts for each of the proteins. Dashed lines on the BioGRID data are for “yeast two hybrid”, “colocalization” or “enzymatic activity” annotations in BioGRID; continuous lines are for co-IP coupled to mass spectrometry or to immunoblotting, as well as for co-crystal structures. The two baits, TRAF3 and USO1/p115 are shown as larger nodes. The previously known TRAF3 interactor is shown in pink. New TRAF3 interactors USO1/p115 and SEC16A are shown in orange. * Stark C, Breitkreutz BJ, Chatr-Aryamontri A, Boucher L, Oughtred R, Livstone MS, Nixon J, Van Auken K, Wang X, Shi X, Reguly T, Rust JM, Winter A, Dolinski K, Tyers M. (2010) The BioGRID Interaction Database: 2011 update. Nucleic Acids Res. 39: D698-704; **Shannon, P. et al. (2003) Cytoscape: a software environment for integrated models of biomolecular interaction networks. Genome Res 13, 2498–2504. (**C–D**) Co-immunoprecipitation experiments showing the association of FLAG-TRAF3, but not TRAF2 or TRAF6, with Myc-p115 or EGFP-Sec16A immunocomplexes.
(TIF)

Figure S2 Selective colocalization of TRAF3 with components of the ER-to-Golgi vesicular pathway. (**A**) Confocal microscopy analysis of HeLa cells transfected with FLAG-tagged TRAF2 (panel 1), TRAF3 (panel 2) or TRAF6 (panel 3). The Golgi apparatus was labeled with an anti-GM130 antibody. (**B**) Colocalization of FLAG-TRAF2 (panel 1), FLAG-TRAF3 (panel 2) or FLAG-TRAF6 (panel 3) with endogenous Sec16A. (**C**) Colocalization of FLAG-TRAF2 (panel 1), FLAG-TRAF3 (panel 2) or FLAG-TRAF6 (panel 3) with Myc-p115.
(TIF)

Figure S3 Microtubule depolarization affects the perinuclear localization of TRAF3. HeLa cells were transfected with FLAG-TRAF3 (panels 1, 2 and 4) or FLAG-TRAF3 and Myc-p115 (panels 3 and 5) and treated with dimethylsulfoxyde (panel 1), 5 µg/ml of nocodazole for 2 h at 37°C (panels 2 and 3) or 5 µg/ml of BFA for 1 h at 37°C (panels 4 and 5) before confocal microscopy analyses. Bars represent 10 µm. One of two independent experiments with similar results is shown.
(TIF)

Figure S4 Silencing of Sec16A and p115 disrupts TRAF3 localization. (**A**) HeLa cells were transfected with 40 nM nonsilencing RNA duplexes (panels 1 and 3) or 40 nM siRNA duplexes that specifically target Sec16A (panels 2 and 4). At 72 h post-transfection, the cells were stained for endogenous GM130, Sec16A, TRAF3, and the nucleus (DAPI). Arrows in panel 2 indicate the silencing effects of the Sec16A siRNA duplexes on the expression pattern of Sec16A and the cellular distribution of GM130. Arrows in panel 4 demonstrate that in the absence of Sec16A, TRAF3 no longer colocalizes with the cis-Golgi marker GM130. Bars represent 5 µm. (**B**) HeLa cells were transfected with 40 nM nonsilencing RNA duplexes (panel 1) or 40 nM siRNA duplexes that specifically targets p115 (panel 2). At 72 h post-transfection, the cells were stained for endogenous TRAF3, p115, and the nucleus (DAPI). The arrows indicate the silencing effect of the p115 siRNA duplexes on the expression pattern of p115 and the cellular distribution of TRAF3. Bars represent 5 µm. One of three independent experiments with similar results is shown.
(TIF)

Figure S5 COPI/COPII-vesicular retention of the TRAF3-AKKFF mutant affects its extraction efficiency as well as interaction with MAVS. 293T cells were co-transfected with Myc-MAVS and the indicated FLAG-TRAF3 constructs (wtTRAF3 or TRAF3-AKKFF). 24 h post-transfection, whole cell extracts were prepared using 1% Triton X-100 or RIPA lysis buffers as indicated. Cellular extracts were then subjected to immunoprecipitation using anti-FLAG antibodies or used in Western blot analysis (Input). Following multiples washing steps, immunoprecipitated proteins were then subjected to Western blot analysis using the indicated antibodies. Interestingly, using a soft lysis condition (1% Triton X-100), we were not able to extract the same amount of the two TRAF3 populations in the IP and in the INPUT (left panels), most likely due to the ability of the TRAF3-AKKFF mutant to be retained in the rich vesicular COPI/COPII environment. On the other hand, the use of a RIPA buffer helped the extraction of the TRAF3-AKKFF mutant from its vesicular-rich environment (right panels). However under these conditions, we might have disrupted the COPI/COPII vesicles, releasing the TRAF3-AKKFF mutant into the cell lysate and allowing its interaction with MAVS. Nonetheless, a lower amount of the TRAF3-AKKFF mutant was detected in the Myc-MAVS immunocomplex compared to wtTRAF3 (right panels).
(TIF)

Figure S6 Mild expression of Sec16A and p115 in Hec1B cells increases activation of ISG56 and NF-κB promoter. (**A–C**) Hec1B cells were co-transfected with pGL3-ISG56-luciferase reporter gene, 125 ng of empty vector, Myc-p115 or Sec16A and 15 ng of His-TRIF (**A**), 15 ng of FLAG-MAVS (**B**) or 100 ng of FLAG-TBK1 (**C**). (**D–F**) Hec1B cells were co-transfected with pGL3-NF-κB-luciferase reporter gene together with 125 ng of empty vector, Myc-p115 or Sec16A and 60 ng of His-TRIF (**D**), 60 ng of FLAG-MAVS (**E**) or 200 ng of FLAG-TBK1 (**F**). Data represent the fold-activation over the corresponding vector control. Each value represents the mean +/− S.D. of triplicate determinations. The data are representative of at least four different experiments with similar results.
(TIF)

Figure S7 Overexpression of p115 or Sec16A in highly transfectable cell lines inhibited TRAF3-dependent transcriptional activation as depletion of Sec16A or p115. (**A–C**) 293T cells were co-transfected with pGL3-IFNβ luciferase reporter gene, plus increasing amounts of indicated plasmids along with 15 ng of His-TRIF (**A**), 15 ng of FLAG-ΔRIGi (**B**) or 15 ng of FLAG-MAVS (**C**). (**D**) 293T cells were co-transfected with pGL3-IFNβ luciferase reporter gene, increasing amount of FLAG-TRAF3, 15 ng of FLAG-MAVS or empty vector and 70 ng of Myc-p115 or EGFP-Sec16A (**D**). (**E–F**) 293T cells were co-transfected with pGL3-ISRE luciferase reporter gene plus increasing amounts of indicated plasmids along with 100 ng of FLAG-TBK1 (**E**) or 15 ng of FLAG-IRF3 5D (**F**). Relative luciferase activity was measured as described in Materials and Methods. Data are representative of at least four different experiments with similar results.
(TIF)
