# Supplementary figures and images for: Proteomic Profiling of the TRAF3 Interactome Network Reveals a New Role for the ER-to-Golgi Transport Compartments in Innate Immunity
Source: PLoS Pathog. 2012 Jul 5;8(7):e1002747. doi: 10.1371/journal.ppat.1002747 (PMC3390413; doi:10.1371/journal.ppat.1002747)

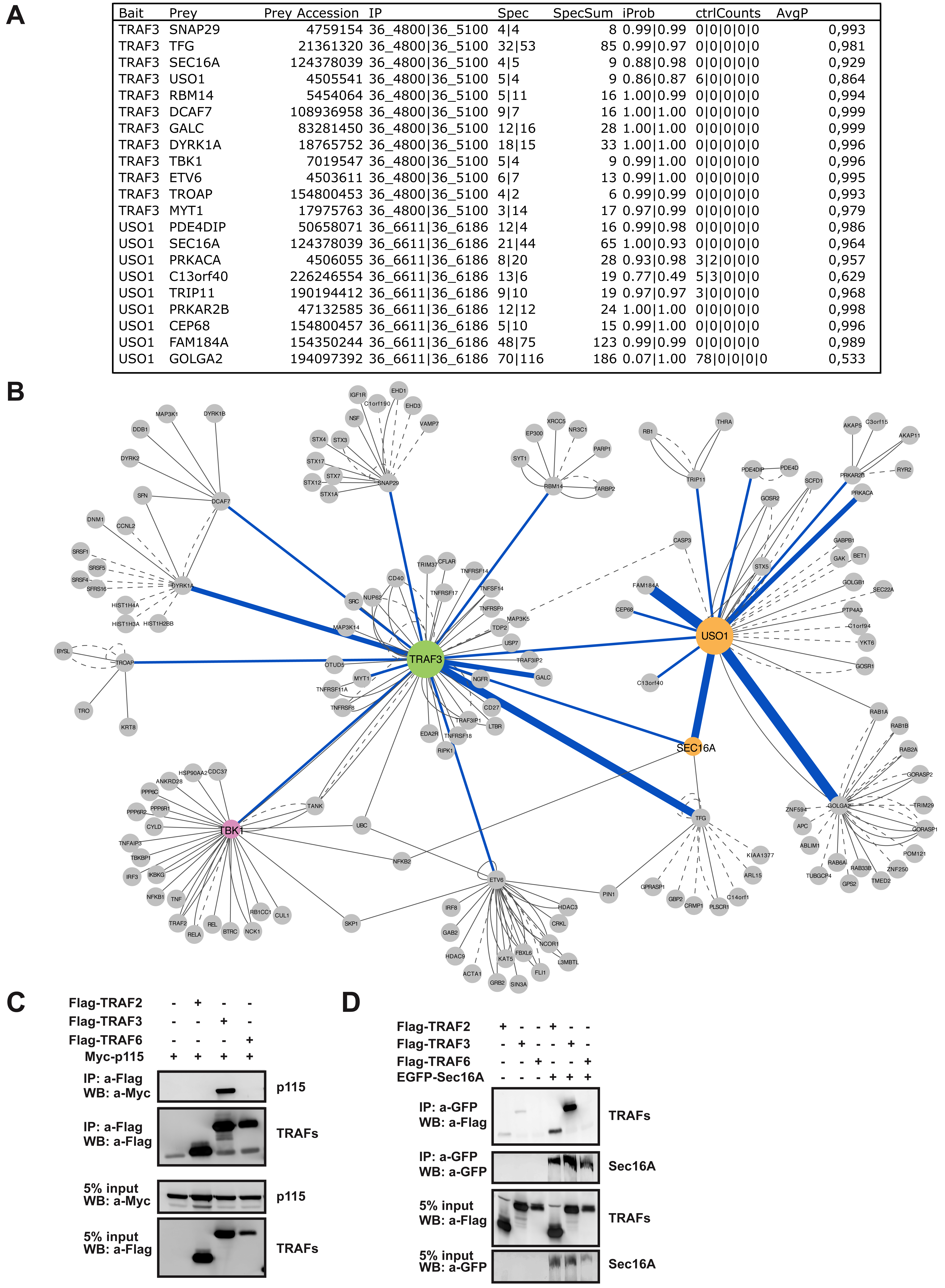

Supplement: Figure S1 — The TRAF3 interactome network. (A) AP-MS data with ≥0.5 AvgP SAINT value. Indicated baits and prey (HUGO gene names; USO1 is the gene name for p115) are listed, alongside the accession number (protein NCBI gi) for the prey, and SAINT output data. Columns are as follows («|» is a delimiter for biological replicates): «IP» are unique identifiers for the experiment in the ProHits database; «Spec» are the spectral counts in each individual experiment; «SpecSum» is the sum of the spectral counts across all analyses. «iProb» is the initial probability in an individual experiment; «crtlCounts» are the spectral counts in five virtual controls, as defined in Methods; «AvgP» is the average of the individual probabilities. The following proteins passed the SAINT threshold filter but were excluded from further analysis, based on high-frequency of detection in FLAG AP/MS analysis from HEK293 cells: EWSR1, FUS, HNRNPC, TUBB2C, HSPA9, HNRNPM, HNRNPH1, ABCA13 and CEP290. (B) Overlay of the filtered mass spectrometry data with literature-curated interactions (as reported in BioGRID version 3.1.76*). Data is visualized in Cytoscape**. The blue colored edges are from the mass spectrometry data in Figure S1A; the grey from literature-curated interactions. The thickness of the blue edges corresponds to the number of spectral counts for each of the proteins. Dashed lines on the BioGRID data are for “yeast two hybrid”, “colocalization” or “enzymatic activity” annotations in BioGRID; continuous lines are for co-IP coupled to mass spectrometry or to immunoblotting, as well as for co-crystal structures. The two baits, TRAF3 and USO1/p115 are shown as larger nodes. The previously known TRAF3 interactor is shown in pink. New TRAF3 interactors USO1/p115 and SEC16A are shown in orange. * Stark C, Breitkreutz BJ, Chatr-Aryamontri A, Boucher L, Oughtred R, Livstone MS, Nixon J, Van Auken K, Wang X, Shi X, Reguly T, Rust JM, Winter A, Dolinski K, Tyers M. (2010) The BioGRID Interaction Database: 20 [file ppat.1002747.s001.tif]

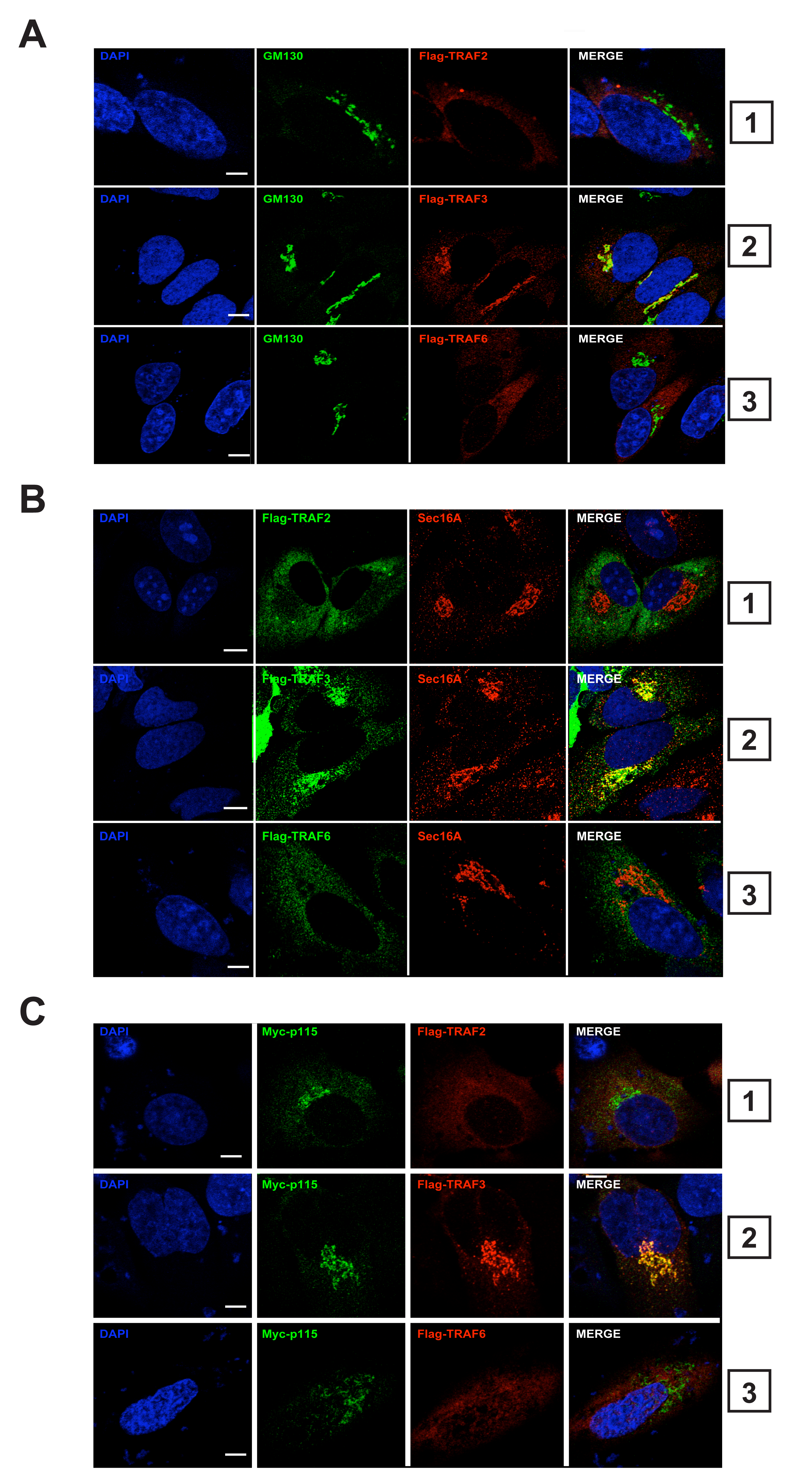

Supplement: Figure S2 — Selective colocalization of TRAF3 with components of the ER-to-Golgi vesicular pathway. (A) Confocal microscopy analysis of HeLa cells transfected with FLAG-tagged TRAF2 (panel 1), TRAF3 (panel 2) or TRAF6 (panel 3). The Golgi apparatus was labeled with an anti-GM130 antibody. (B) Colocalization of FLAG-TRAF2 (panel 1), FLAG-TRAF3 (panel 2) or FLAG-TRAF6 (panel 3) with endogenous Sec16A. (C) Colocalization of FLAG-TRAF2 (panel 1), FLAG-TRAF3 (panel 2) or FLAG-TRAF6 (panel 3) with Myc-p115. (TIF) [file ppat.1002747.s002.tif]

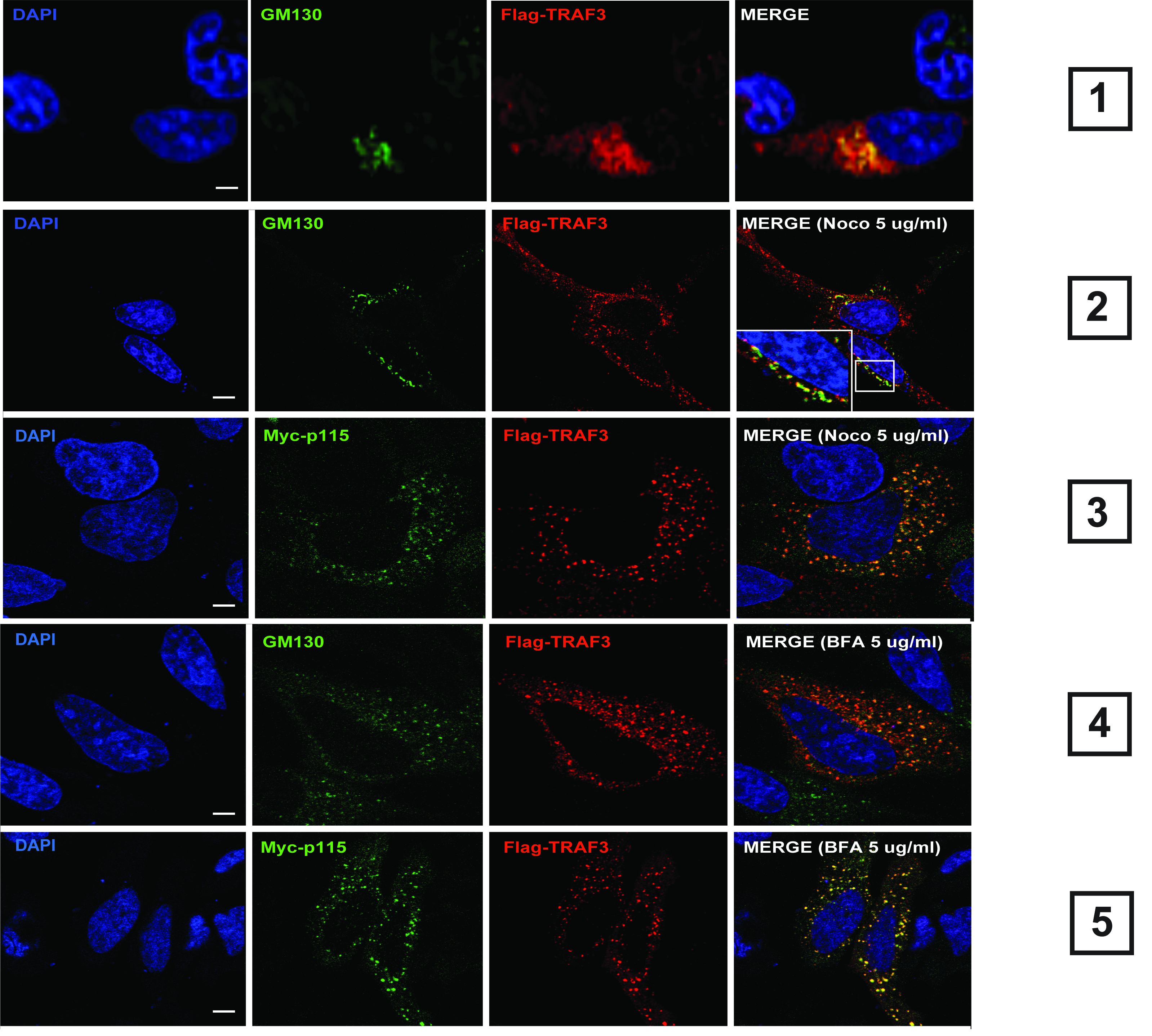

Supplement: Figure S3 — Microtubule depolarization affects the perinuclear localization of TRAF3. HeLa cells were transfected with FLAG-TRAF3 (panels 1, 2 and 4) or FLAG-TRAF3 and Myc-p115 (panels 3 and 5) and treated with dimethylsulfoxyde (panel 1), 5 µg/ml of nocodazole for 2 h at 37°C (panels 2 and 3) or 5 µg/ml of BFA for 1 h at 37°C (panels 4 and 5) before confocal microscopy analyses. Bars represent 10 µm. One of two independent experiments with similar results is shown. (TIF) [file ppat.1002747.s003.tif]

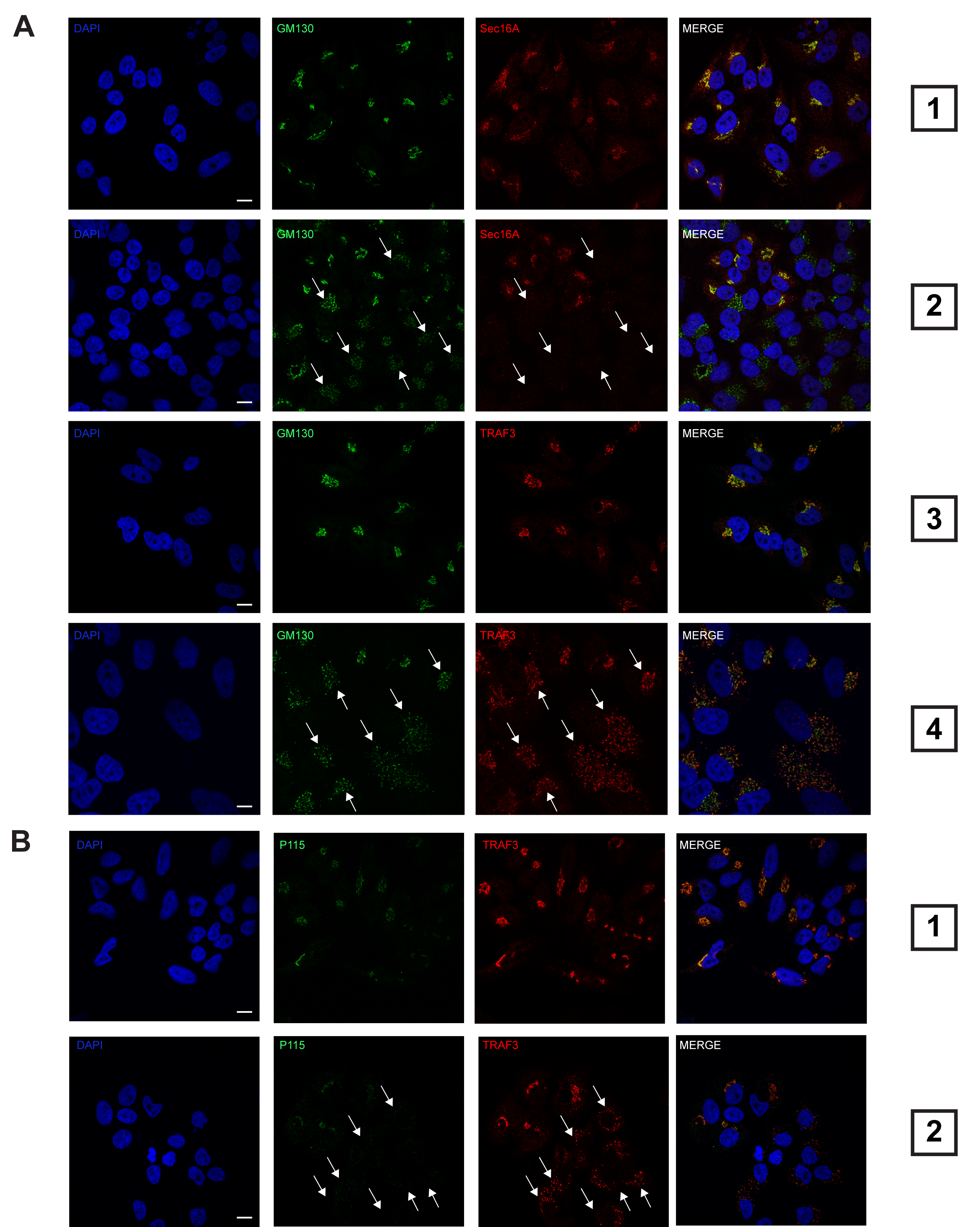

Supplement: Figure S4 — Silencing of Sec16A and p115 disrupts TRAF3 localization. (A) HeLa cells were transfected with 40 nM nonsilencing RNA duplexes (panels 1 and 3) or 40 nM siRNA duplexes that specifically target Sec16A (panels 2 and 4). At 72 h post-transfection, the cells were stained for endogenous GM130, Sec16A, TRAF3, and the nucleus (DAPI). Arrows in panel 2 indicate the silencing effects of the Sec16A siRNA duplexes on the expression pattern of Sec16A and the cellular distribution of GM130. Arrows in panel 4 demonstrate that in the absence of Sec16A, TRAF3 no longer colocalizes with the cis-Golgi marker GM130. Bars represent 5 µm. (B) HeLa cells were transfected with 40 nM nonsilencing RNA duplexes (panel 1) or 40 nM siRNA duplexes that specifically targets p115 (panel 2). At 72 h post-transfection, the cells were stained for endogenous TRAF3, p115, and the nucleus (DAPI). The arrows indicate the silencing effect of the p115 siRNA duplexes on the expression pattern of p115 and the cellular distribution of TRAF3. Bars represent 5 µm. One of three independent experiments with similar results is shown. (TIF) [file ppat.1002747.s004.tif]

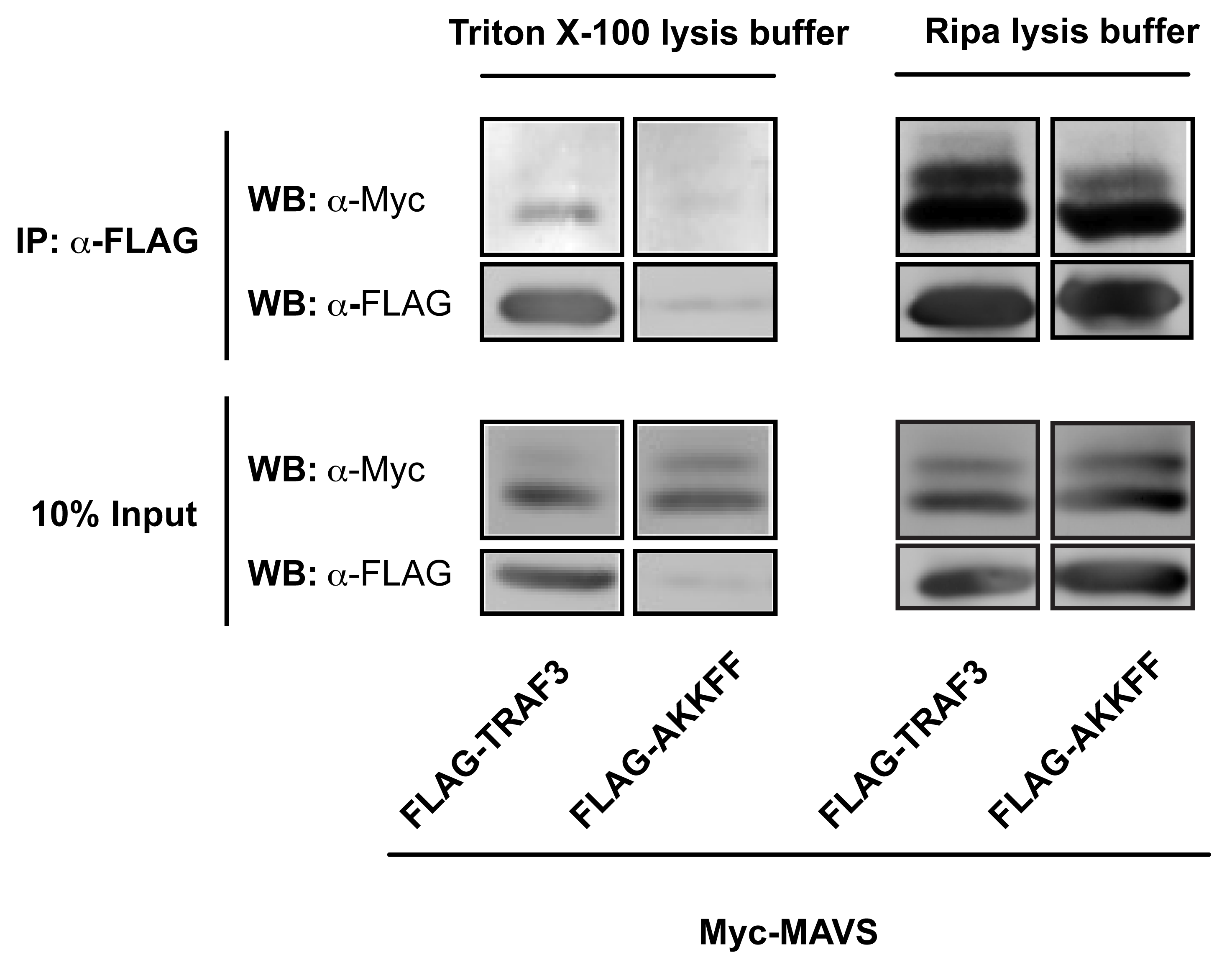

Supplement: Figure S5 — COPI/COPII-vesicular retention of the TRAF3-AKKFF mutant affects its extraction efficiency as well as interaction with MAVS. 293T cells were co-transfected with Myc-MAVS and the indicated FLAG-TRAF3 constructs (wtTRAF3 or TRAF3-AKKFF). 24 h post-transfection, whole cell extracts were prepared using 1% Triton X-100 or RIPA lysis buffers as indicated. Cellular extracts were then subjected to immunoprecipitation using anti-FLAG antibodies or used in Western blot analysis (Input). Following multiples washing steps, immunoprecipitated proteins were then subjected to Western blot analysis using the indicated antibodies. Interestingly, using a soft lysis condition (1% Triton X-100), we were not able to extract the same amount of the two TRAF3 populations in the IP and in the INPUT (left panels), most likely due to the ability of the TRAF3-AKKFF mutant to be retained in the rich vesicular COPI/COPII environment. On the other hand, the use of a RIPA buffer helped the extraction of the TRAF3-AKKFF mutant from its vesicular-rich environment (right panels). However under these conditions, we might have disrupted the COPI/COPII vesicles, releasing the TRAF3-AKKFF mutant into the cell lysate and allowing its interaction with MAVS. Nonetheless, a lower amount of the TRAF3-AKKFF mutant was detected in the Myc-MAVS immunocomplex compared to wtTRAF3 (right panels). (TIF) [file ppat.1002747.s005.tif]

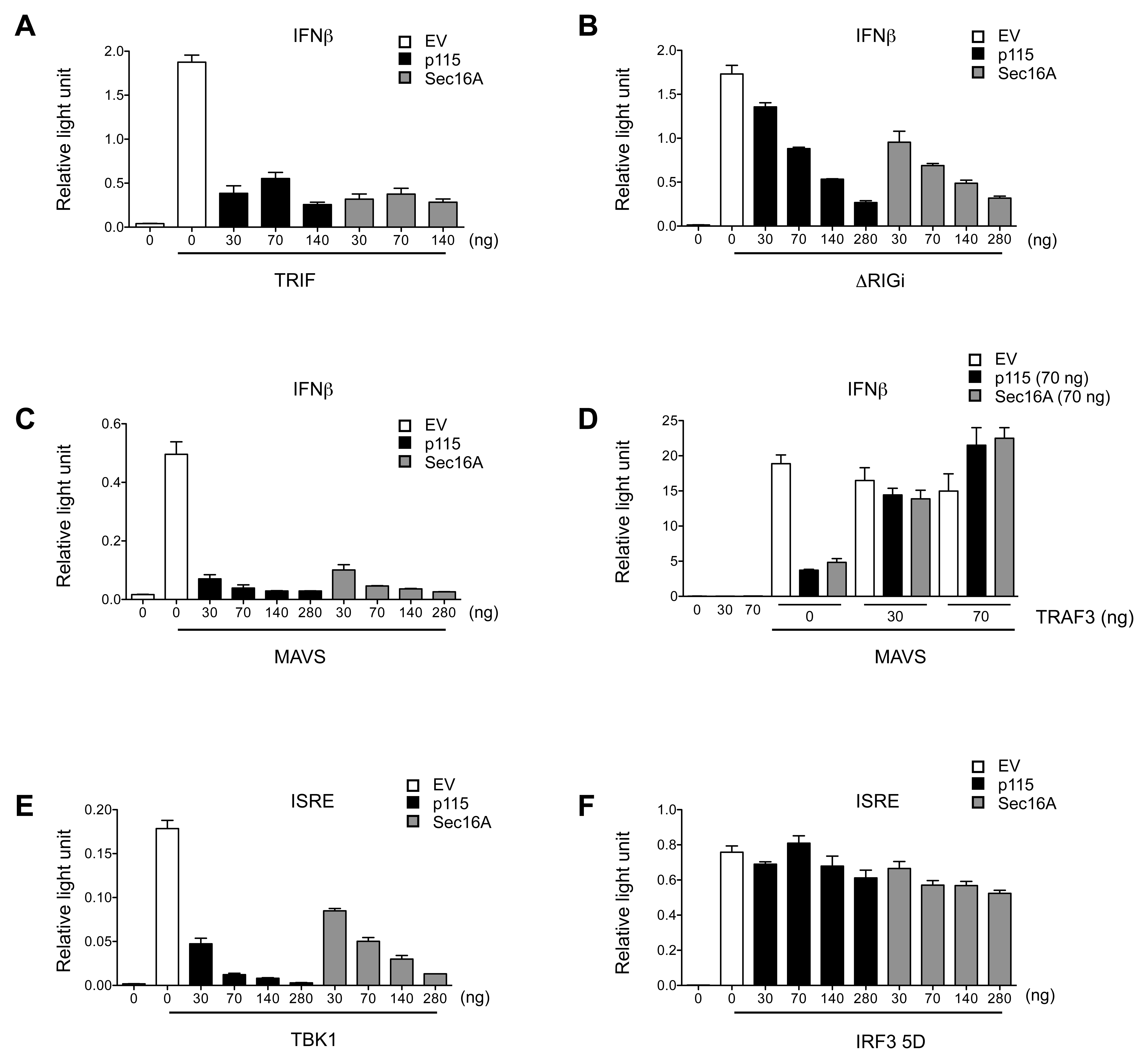

Supplement: Figure S7 — Overexpression of p115 or Sec16A in highly transfectable cell lines inhibited TRAF3-dependent transcriptional activation as depletion of Sec16A or p115. (A–C) 293T cells were co-transfected with pGL3-IFNβ luciferase reporter gene, plus increasing amounts of indicated plasmids along with 15 ng of His-TRIF (A), 15 ng of FLAG-ΔRIGi (B) or 15 ng of FLAG-MAVS (C). (D) 293T cells were co-transfected with pGL3-IFNβ luciferase reporter gene, increasing amount of FLAG-TRAF3, 15 ng of FLAG-MAVS or empty vector and 70 ng of Myc-p115 or EGFP-Sec16A (D). (E–F) 293T cells were co-transfected with pGL3-ISRE luciferase reporter gene plus increasing amounts of indicated plasmids along with 100 ng of FLAG-TBK1 (E) or 15 ng of FLAG-IRF3 5D (F). Relative luciferase activity was measured as described in Materials and Methods. Data are representative of at least four different experiments with similar results. (TIF) [file ppat.1002747.s007.tif]
